# Mechanisms of Tebuconazole Adsorption in Profiles of Mineral Soils

**DOI:** 10.3390/molecules26164728

**Published:** 2021-08-04

**Authors:** Marcin Siek, Tadeusz Paszko, Maria Jerzykiewicz, Joanna Matysiak, Urszula Wojcieszek

**Affiliations:** 1 Department of Chemistry, University of Life Sciences, Akademicka 13, 20-950 Lublin, Poland; maciek.siek@wp.pl (M.S.); joanna.matysiak@up.lublin.pl (J.M.); urszula.wojcieszek@up.lublin.pl (U.W.); 2 Faculty of Chemistry, University of Wrocław, F. Joliot-Curie 14, 50-383 Wrocław, Poland; maria.jerzykiewicz@uwr.edu.pl

**Keywords:** tebuconazole, adsorption, organic matter fractions, soil acidity, FT-IR

## Abstract

The study attempted to identify the soil components and the principal adsorption mechanisms that bind tebuconazole in mineral soils. The *K_F_* values of the Freundlich isotherm determined in 18 soils from six soil profiles in batch experiments after 96 h of shaking ranged from 1.11 to 16.85 μg^1^^−*1/n*^ (mL)*^1/n^* g^−1^, and the exponent *1/n* values from 0.74 to 1.04. The adsorption of tebuconazole was inversely correlated with the soil pH. Both neutral and protonated forms of this organic base were adsorbed mainly on the fraction of humins. The adsorption of the protonated form increased in the presence of hydrogen cations adsorbed in the soil sorption sites. Fourier transform infrared spectroscopy coupled with the molecular modeling studies and partial least squares regression analysis indicated that the tebuconazole molecule is bound in the organic matter through the formation of hydrogen bonds as well as hydrophobic and π–π interactions. Ion exchange was one of the adsorption mechanisms of the protonated form of this fungicide. The created mathematical model, assuming that both forms of tebuconazole are adsorbed on the organic matter and adsorption of the protonated form is affected by the potential acidity, described its adsorption in soils well.

## 1. Introduction

Tebuconazole (TB) ((RS)-1-(4-chlorophenyl)-4,4-dimethyl-3-(1*H*-1,2,4-triazol-1-ylmethyl)-pentan-3-ol) is one of the most widely used systemic fungicide in agriculture. It belongs to the class of azole fungicides, which exhibit diverse acidic-basic characteristics [1,2,3]. It acts mainly by inhibiting demethylation in the biosynthesis of fungal sterols [4] and is effective against various foliar diseases in cereals, sugarcane, grapes, peanuts, and many vegetables [3]. On the other hand, TB is believed to cause endocrine disruption and to affect the reproduction system and poses a threat to the environment. Therefore, it is considered a candidate for substitution in the European Union (EU) [5,6]. In some European countries, the concentrations of TB detected in the surface water are many folds higher than the limit allowed by the EU [7,8]. Moreover, this is one of the seven most common soil contaminants in the EU, being found in >10% of the soil samples [9]. In the subsoils, particularly those with a small content of soil organic matter (SOM), TB degradation can be very slow (half-lives up to 3904 days) [8]. Therefore, it can be suspected that in such profiles TB can move to the subsoil and even reaches the groundwater.

The small solubility (36–38 mg/L of water at 20 °C and pH 5.3–9.4) and large hydrophobicity of the TB molecule (log P = 3.7 at 20 °C and pH 7; P is the octanol–water partition coefficient) are associated with its moderate-to-strong adsorption in soils [3,4,10,11]. For example, the EU dossier presented that the values of adsorption coefficient of the Freundlich isotherm (*K_F_*) for nine soils with pH of 5.2–7.4 were in the range of 1.52–16.39, and the values of the Freundlich exponent (*1*/*n*) were in the range of 0.711–1.204 [4]. A study by Mosquera-Vivas et al. showed that only a small part of TB adsorbed in soils exhibited the ability of desorption (1.5–30.1%) [2]. A study by Čadková et al. indicated that adsorption of TB in the separate soil components decreased in the following order: humic acids (HA) > ferrihydrite > birnessite > goethide [12]. Similarly, the recent study by Škulcová et al. showed that TB adsorption decreased in the following order: peat > garden soil > HA >> kaolin clay > quartz sand [1]. Therefore, it is assumed that in soils TB is adsorbed predominantly by SOM, and to a much lesser extent, by inorganic soil components, mainly clay minerals [1,2,13,14]. It was suspected that the adsorption of TB in soils is somehow related to their cation exchange capacity [1] or exchange acidity [2] as well as the Cu content in soils (TB and other triazole fungicides form coordination compounds with Cu^2+^ [13,15]). However, so far no study has clearly indicated which of the inorganic components absorb TB and under what conditions.

The previous studies have focused primarily on the role of SOM in the adsorption of TB. Mosquera-Vivas et al. examined the adsorption of TB in Colombian top and subsoils [2]. They suggested that adsorption was associated with the aromatic rings and carbonyl C=O groups of SOM. Čadková et al. studied the adsorption of TB in three soils with fractions of SOM determined using the extraction methods [13]. The authors observed that the order of adsorption was, in general, consistent with the HA content in soils, and therefore they suggested that this fraction can play the dominant role in the adsorption of the fungicide. Mal’tseva et al. and Tchaikovskaya et al. postulated that the triazole ring of TB protonated at a low pH enters into donor–acceptor interactions with the ionized functional groups of HA [16,17].

The pH-dependent adsorption of TB in soils was neglected for a long time. It was believed that TB is a weak base that can be only completely protonated in nonaqueous systems in the presence of very strong acids [4,13]. Barely in the last decade it was found by experimental study that the protonated form of TB exists in aqueous solutions, and the *pK_a_* of TB ranges from 5.0 to 5.03 at 25 °C [3,13] (*pK_a_* = −log *K_a_*, *K_a_* is the equilibrium constant between the protonated and molecular TB). The dependence of TB adsorption on the soil pH was not observed in the previous studies [2,3,4,10,13] but the examined soils most often have a near-neutral pH. In the available literature, a clear inverse relationship between the TB adsorption and pH was observed only during the adsorption of this compound on Fe and Mn oxides by Čadková et al. [12], and in soils by Badawi et al. and Bošković et al. [5,14].

The first tier of modeling of pesticide leaching into the groundwater used in the regulatory models is based on the assumption that pesticides are sorbed only by SOM (*K_F_*_OC_ concept is used; *K_FOC_* = 100∙*K_F_*/*OC* where *OC* is a soil organic carbon content (%)). It is well-known that such an assumption can introduce a significant systemic bias into the model predictions as the contribution of inorganic soil components to the total sorption is neglected [18,19]. The insufficient number of compound-specific pesticide sorption studies in soil profiles is one of the main reasons for the lack of an idea how to improve the adsorption models implemented in the pesticide leaching programs in order to take into account sorption by a few soil components and (if justified) the effect of soil pH [18]. The power law model has been used recently to improve the *K_F_*_OC_ concept by Jarvis [18]. However, its parametrization was in some cases difficult because the available dataset of adsorption data in the subsoils is still small.

Thus, the general aim of the present laboratory study was to broaden the scope of knowledge related to the adsorption mechanisms of basic pesticides, which is necessary to modify the abovementioned adsorption models. In order to do this, the effect of 26 soil properties on TB adsorption in 18 soils was elucidated. Moreover, the Fourier transform infrared (FT-IR) spectroscopy spectra of soils were examined. To the best of our knowledge this is the first study which presents readable FT-IR spectra for the subsoils with a very small initial OC content, and one of a few studies which examine the relationship between the functional groups of SOM assessed by means of FT-IR and pesticide adsorption parameters [20,21]. The specific objectives of this study were: (i) to determine the adsorption parameters of TB for further modeling of its leaching in Polish mineral soils, (ii) to assess the principal organic and inorganic components of soil contributing to its adsorption and create a reliable adsorption model, and (iii) to identify the principal mechanisms governing its adsorption at low and high soil pH.

## 2. Results

### 2.1. Preliminary Analysis—Adsorption Isotherms

The soils examined in this study, classified as Arenosols, Luvisols, and Chernozems [22], cover 3.6%, 14.7%, and 1.9% of the total land surface of the EU, respectively [23]. In Poland Arenosols and Luvisols cover more than half of the total area of arable soils, and Chernozems 1% [8,24] (Figure S1 and Table S1 in the Supplementary Materials). In the analysis of adsorption isotherms of pesticides in soils the Freundlich model is most often used; its application is proposed, e.g., by the OECD guideline 106 [25]. The Freundlich model fitted well the adsorption isotherms (Figure S2 and Table S2 in the Supplementary Materials), while the fit of the linear model (Freundlich model with the assumption that *1/n =* 1) was a bit worse. The determination coefficient (R^2^) values were in the ranges of 0.936–0.994, and 0.897–0.991, respectively.

According to the classification system proposed by Giles et al., the obtained isotherms were of L (*1/n* < 1) or C (*1/n* = 1) type [26]. The L-isotherms are found when the number of adsorbent sites decreases as the solute concentration increases. This type of isotherm is usually observed when adsorption approaches equilibrium slowly, and isotherms become increasingly nonlinear with time [27]. In turn, a constant partition of the solute between the bulk solution and the adsorbent surface (C-isotherm) may occur when the low-solubility (hydrophobic) solute is attracted more by certain porous adsorbent regions than water [26]. The C-type isotherms were found in eight soils and the L-isotherms in ten soils (Table S2 in the Supplementary Materials).

**Figure 1:**
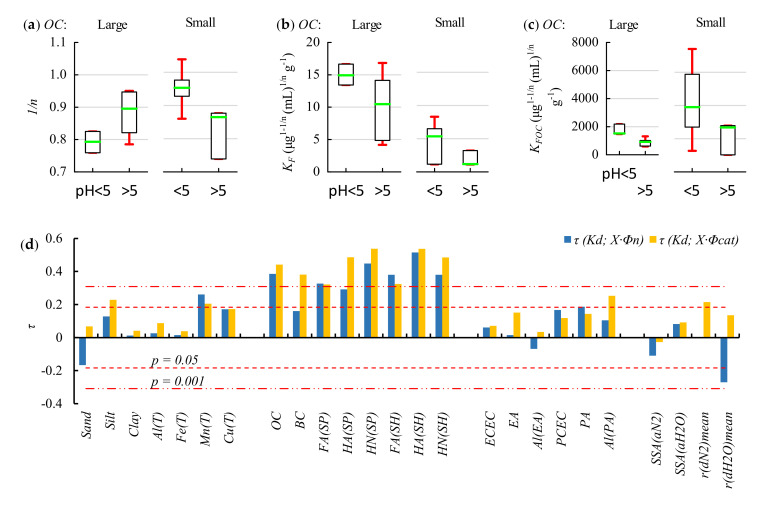
Result of **TB** adsorption experiments. (**a**) Relationships between *1*/*n*, (**b**) *K_F_*, (**c**) *K_FOC_* and pH in the soils with large (0.48–1.92%, n = 9) and small (0.03–0.31%, n = 9) *OC* contents. The box plots show the minimum, first quartile, median, third quartile, and maximum. (**d**) Kendall’s rank correlations (*τ*) between *K_d_* and 25 soil properties (for definitions see Section 3.1) multiplied by *Φ_n_* and *Φ_cat_*.

Figure 1a shows that the largest *1*/*n* values were observed in soils having small SOM content and pH < 5. The post-hoc Tukey’s test of the two-factor analysis of variance indicated at pH < 5 a significant difference (*p* = 0.04) existed between the means of *1*/*n* values for soils with large and small *OC* contents. This signalized the possibility of more than one mechanism of adsorption of the TB protonated form or more than one soil component participating in its adsorption. The minimum, median, and maximum *1*/*n* values available in the literature for mineral soils [2,10,13,28,29,30] were 0.53, 0.89, and 1.08, respectively. Thus, the *1*/*n* values presented in Figure 1a are within the range of values determined for TB by other authors. The obtained *K_F_* and *K_FOC_* values (Figure 1b,c) clearly indicated that the adsorption process was pH-dependent—adsorption was larger at pH < *pK_a_* of TB, where its protonated form dominated, and smaller at pH > *pK_a_*, where its neutral form was predominant. The contribution of components other than SOM in adsorption at pH < 5 is proven by very large *K_FOC_* values in subsoils with small SOM content (range: 1266–7406 μg^1^^−*1/n*^ (mL)*^1/n^* g^−1^) in comparison to the *K_FOC_* values determined in topsoils and subsoils with large SOM content (range: 1026–2784 μg^1^^−*1/n*^ (mL)*^1/n^* g^−1^). 

Adsorption of TB in soils from different depths has been examined so far only in a few studies. In the study by Mosquera-Vivas et al. the *K_FOC_* values obtained for subsoils (40–50 cm) were 1.3- to 3.5-fold larger than those determined for topsoils (0–10 cm) [2]. In turn, no significant differences were noted in the study by Badawi et al. for soils from depths up to 12 cm [14]. The *K_FOC_* values obtained for topsoils by other authors were in the range of 287–5172 μg^1^^−*1/n*^ (mL)*^1/n^* g^−1^ [2,10,13,28,29,30], while the values obtained in this study were in the range of 877–2193 μg^1^^−*1/n*^ (mL)*^1/n^* g^−1^.

In summary, the analysis of the adsorption isotherms indicated that TB adsorption was a pH-dependent process and that at pH < 5 there can be expected to be a few adsorption mechanisms, including adsorption on inorganic soil components. The potential contribution of inorganic soil components to adsorption suggested that using organic matter-normalized adsorption coefficients may not be a proper way for describing the pH-dependent adsorption processes of the fungicide.

### 2.2. Soil Properties Affecting Adsorption—Results of pH-Dependent Adsorption Analyses

#### 2.2.1. pH-Dependent Adsorption Model

Assuming that the neutral form of the basic pesticide is adsorbed on SOM and the protonated form on SOM and on *X* soil component (e.g., clay minerals), the pH-dependent adsorption can be described as follows [31,32]:

(1) $$K_{d}=\kappa_{OC\left(n\right)}\cdot OC\cdot \Phi_{n}+\kappa_{OC\left(cat\right)}\cdot OC\cdot \Phi_{cat}+\kappa_{X\left(cat\right)}\cdot X\cdot \Phi_{cat}$$

where *K_d_* (mL/g) is the distribution coefficient for adsorption, and $\kappa_{OC\left(n\right)}$,$\;\kappa_{OC\left(cat\right)}$, and $\kappa_{X\left(cat\right)}$ are the regression coefficients for adsorption of neutral and cationic forms of the fungicide on SOM, and the cationic form on *X* component, respectively. The neutral (Φ*_n_*) and protonated (Φ*_cat_*) fractions of the pesticide, which describe the nonlinear relationship between *K_d_* and independent variables and are dependent on the *pK_a_* value and the pH in the soil suspension, are calculated using the Henderson–Hasselbalch equation:

(2) Φn=11+10pKa−pH

(3) Φcat=11+10pH−pKa

#### 2.2.2. Correlation Analysis

Initial exploratory data analysis was performed based on the analysis of correlation coefficients. For this, *K_d_* values, calculated separately for the triplicate soil samples with an initial TB concentration of 2.0 μg/mL, were used. Besides the simple correlation analysis presented in Table S3 in the Supplementary Materials, the correlations between the *K_d_* and *X* soil component/property multiplied by *Φ_n_* or by *Φ_cat_* were calculated (the pH values measured in batch experiments were used, as well as the TB *pK_a_* of 5.0 [3]). This is because the native pH values of the majority of examined soils were in the range of TB *pK_a_* ± 2. In this pH range, the relationship between *K_d_* and any of the soil properties influencing the adsorption is affected by pH (Equations (1)–(3)). The correlations calculated as above exclude the pH effect. Moreover, they can be treated as the initial estimates of the affinity of individual soil components to the protonated and neutral form of TB.

Kendall’s rank correlations (*τ*) were used because they do not require normal distribution of the analyzed variables. It is noticeable that the correlations of *K_d_* with SOM-associated variables were generally the highest (Figure 1d). Correlations with variables such as silt fraction (*Silt* (%)), organic complexes + poorly crystallized oxides of Mn dissolved with Tamm’s solution [33] (*Mn*(*T*) (g/kg)), exchangeable + occluded on Fe and Mn oxides + complexed in SOM Cu dissolved with Tamm’s solution [33] (*Cu*(*T*) (mg/kg)), exchangeable acidity (*EA* (cmol(+)/kg)), potential acidity (*PA* (cmol(+)/kg)), potential cation exchange capacity (*PCEC* (cmol(+)/kg)), Al cations contributing to *PA* (*Al*(*PA*) (cmol(+)/kg)), and mean pore radius from the N_2_ desorption isotherms (*r*(*dN*_2_)*mean* (nm)) were positive but insignificant or significant only at *p* < 0.05. Thus, data presented in Figure 1d indicated the dominant contribution of proxy variables associated with SOM to the adsorption of TB.

#### 2.2.3. Regression Analyses

Next, the adsorption of neutral and protonated forms of TB was analyzed using regression analysis methods. The use of Equation (1) became complicated when SOM fractions were used. The fractions were correlated to each other and were additionally multiplied by Φ*_n_* and Φ*_cat_*. As a result, the independent variables were more strongly correlated with each other than with *K_d_*. To cope with the collinearity of the independent variables, partial least squares regression (PLSR) analysis was used [34,35,36]. Similar to the correlation analysis, the analyzed soil parameters were multiplied by the calculated Φ*_n_* and Φ*_cat_* for each soil sample, and the PLSR was carried out using the transformed variables. For example, for the analysis of sorption in the fulvic acids (*FA*), humic acids (*HA*), and humins (*HN*) determined using 0.1 M sodium hydroxide (SH), the variables denoted as *FA*_(*SH*)*Φn*_, *HA*_(*SH*)*Φn*_, and *HN*_(*SH*)*Φn*_ and *FA*_(*SH*)*Φcat*_, *HA*_(*SH*)*Φcat*_, and *HN*_(*SH*)*Φcat*_ were used. For each analyzed dataset the number of latent vectors (LV) was estimated for the data, which were cross-validated and partitioned into five blocks, assuming that Wold’s *Q*^2^ must exceed 0.0975. The significant independent variables were selected by stepwise elimination of variables with the smallest standardized regression coefficients and variable importance on projection (VIP). The details about variable transformation and PLSR analysis can be found elsewhere [36].

Initially it was assumed that TB is adsorbed on SOM only. The SOM-related variables were divided into three groups—*OC* and black carbon fraction (*BC* (%)); fractions determined using 0.1 M sodium pyrophosphate (SP) (*FA*(*SP*), *HA*(*SP*), and *HN*(*SP*)); and fractions determined using 0.1 M SH (*FA*(*SH*), *HA*(*SH*), and *HN*(*SH*))—and were analyzed separately. The results (Table 1) indicated that adsorption of both forms of TB on *BC* was not significant. The standardized regression coefficients (as well as VIP values) were found to be much lower for *BC* than for *OC-BC* (Equation (4)). Moreover, the R^2^ values obtained for Equations (4) and (5) were practically the same. Soil *BC* (char, charcoal, and soot from crop residue burning, natural fires, or fossil fuel combustion [37]) is a normal component of agricultural soils, and occurs at rates of up to 10% or more. *BC* contains some dissociable carboxyl and hydroxyl groups [38], but exhibits higher affinity to the some of nonionizable organic compounds [37,39]. The results of the PLSR analysis of the adsorption of TB molecule on the SOM fractions (Equations (7) and (9)) were consistent and showed that this form was adsorbed predominantly by *HN*. The *HN* fraction is characterized by the highest degree of polymerization and the lowest content of carboxylic groups, and is the most hydrophobic of all SOM fractions [40]. Therefore, it is likely that this fraction adsorbs the largest amount of TB molecules. The backward regression indicated that the *HN* and, to a lesser extent, *FA* fraction were also responsible for the adsorption of TB cations (it can be noted that the R^2^ values obtained for Equations (7) and (9) are higher than those obtained for Equations (6) and (8)). One of the possible reasons for this was that *HN* was the dominant fraction of SOM in the examined soils (Figure S3 in the Supplementary Materials) and the lower concentration of sorption sites available for TB cations in *HN* (e.g., carboxylic groups) was compensated by its higher amount.

Next, three groups of variables associated with SOM were examined together with 17 variables associated with inorganic components, sorption capacity, and surface properties of soils (Figure 1d). The PLSR analysis indicated that the adsorption of the neutral form of TB was associated with *OC-BC* (Equation (10)), *HN*(*SP*) (Equation (11)), and with *HN*(*SH*) and to a lesser extent with *HA*(*SH*) variables (Equation (12)). Thus, modeling results indicated that TB molecules were sorbed only by SOM, mainly by the *HN* fraction. According to Equations (10)–(12), the variables *OC-BC*, *HN*(*SP*), and *HN*(*SH*) were also the most important variables contributing to the adsorption of protonated TB, and *PA* was the second most important variable (compare the standardized regression coefficient values). Removal of the *PA_Φcat_* variable from Equations (10)–(12) decreased the prediction of *K_d_* variance by 5.3%, 6.9%, and 4.5%, respectively. Performing the nonlinear regression analysis using the variables from Equation (10), i.e.,

(13) $$K_{d}=10.60_{\left(<0.001\right)}\cdot \left(OC-BC\right)\cdot \Phi_{n}+16.41_{\left(<0.001\right)}\cdot \left(OC-BC\right)\cdot \Phi_{cat}+0.80_{\left(<0.001\right)}\cdot PA\cdot \Phi_{cat}$$

(R^2^ = 0.963; values given in brackets in subscript are *p*-values) made it possible to estimate the contribution of the *PA* variable to the estimated *K_d_* values in individual soils. A small contribution of *PA* to *K_d_* (0–14%) was found in the soils with large *OC* (0.48–1.92%) and pH > 5. However, in soils with small *OC* contents (0.03–0.31%) and pH < 5 the contribution was very large (44–80%).

It is commonly assumed that *PA* determined using BaCl_2_-TEA (triethanolamine) solution at pH 8.2 is a measure of acidity resulting from the replacement of hydrogen and aluminum cations from the permanent (i.e., difficult for dissociation and extraction) and the pH-dependent (exchangeable) sites of SOM and clay minerals [41,42]. Applying the model proposed by Curtin and Rostad [42], it was found that the main source of *PA* in SOM is the *FA* fraction (*HA* and *HN* variables were insignificant). The best model (R^2^ = 0.902) was obtained for the *FA*(*SH*) fraction as follows:

(14) $$PA=1.30_{\left(0.003\right)}+\left(8.2-pH\right)\cdot \left(6.76_{\left(<0.001\right)}FA\left(SH\right)+0.08_{\left(<0.001\right)}Clay\right)$$

where pH denotes the pH of native soil. Worse results were obtained after the replacement of *FA*(*SH*) in Equation (14) with *FA*(*SP*) (R^2^ = 0.885), *OC-BC* (R^2^ = 0.826), and *OC* (R^2^ = 0.819). The regression analysis with the assumption that the intercept in Equation (14) is equal to 0 made it possible to calculate that in the topsoils *PA* resulting from the replacement of hydrogen and aluminum cations from the clay minerals was in the range of 11–49% of the total *PA*, while in the subsoils it was in the range of 27–92%.

The correlations between *K_d_* and *Al*(*PA*) shown in Figure 1d are higher than those calculated for *PA*. However, the PLSR analysis did not confirm that *Al*(*PA*) contributed to TB adsorption. Thus, the adsorption of the protonated TB form was associated with hydrogen cations that were adsorbed in permanent and exchangeable sorption sites. A high contribution of clay minerals to *PA* indicates that protonated TB was also adsorbed on this soil component, especially in subsoils. It is worth noting that replacement of the *PA_Φcat_* variable in Equations (10)–(12) with *Clay_Φcat_* resulted in lower R^2^ values, but higher than those determined for Equations (5), (7) and (9). Some contribution of clay minerals in TB adsorption has been suggested by other authors [2,13,14]. Škulcová et al. indicated that the sorption of TB by peat and *HA* was much larger than that of kaolin clay or quartz sand (*K_d_* = 529, 285, 4.56, and 1.25 mL/g, respectively) but there were also differences due to the pH of the adsorbents (4.6, 3.0, 5.7, and 7.1, respectively) [1].

Recently, four different adsorption models were tested in six Colombian soils by Mosquera-Vivas et al. [2]. The authors assumed that TB is only adsorbed on SOM and obtained the best result for the linear model assuming that adsorption is independent of pH. This was most likely due to the fact that the authors used soils with a narrow pH range (5.1–6.0) and a low number of soils. It is worth noting that the assumption that adsorption is pH-independent led to much worse results in our study; for example, after replacing the variables *X_Φn_* and *X_Φcat_* in Equations (10)–(12) with *X* variables and performing simple linear regression, the R^2^ values obtained were in the range of 0.829–0.915.

It is worth noting that the general formula proposed in Equation (1) described well not only the adsorption data for TB (Equation (13)) but also for carbendazim (in this case Equation (1) with assumption that $\kappa_{OC\left(cat\right)}=0$ and *X* = *Clay* can be used). Adsorption of carbendazim, another basic fungicide, was examined in our previous studies [32]. Therefore, we believe that this simple formula is universal enough to also be successfully applied for other basic pesticides, including other basic azole fungicides.

### 2.3. Relationship between FT-IR Characteristics of SOM and Adsorption of TB

The FT-IR spectra of native mineral soils are dominated by bands of mineral matter which overlap the organic matter signal [43]. This is due to the presence of clay minerals and quartz, which gives rise to adsorption bands in the FT-IR spectra and which needs to be removed (at least partially) if spectra of the organic matter of mineral soils are to be recorded. According to the detailed studies by Rumpel et al. the chemical composition of organic matter did not change significantly after the HF treatment, but the FT-IR analysis of organic matter in soils with its low content became possible only after the treatment [43]. Demineralization of soil samples with HF increased their *OC* contents from the initial range of 0.11–1.92% to 1.45–16.15% (Tables S1 and S4 in the Supplementary Materials).

**Figure 2:**
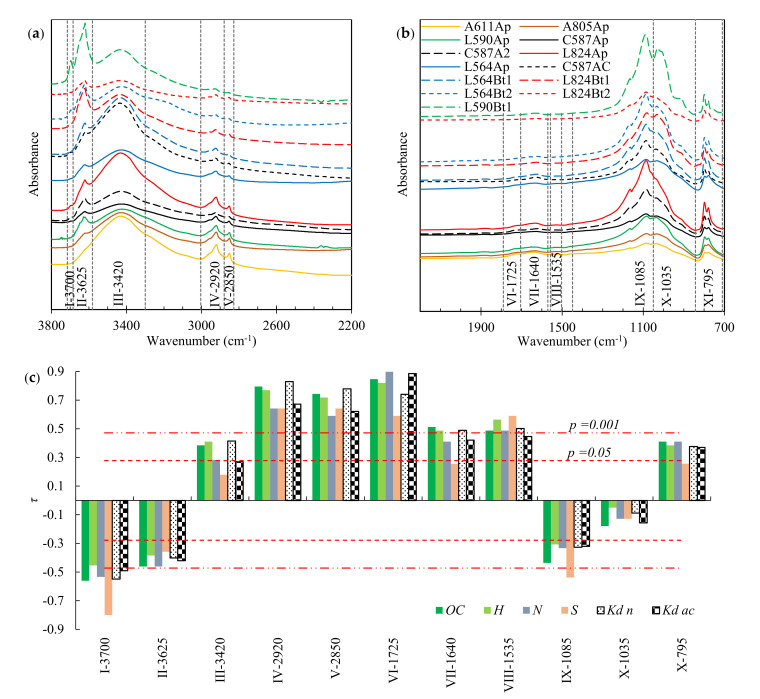
(**a**,**b**) FT-IR spectra of 13 soils altered with HF, shifted vertically to avoid overlapping and arranged in order from the largest (A611Ap) to the smallest (L824Bt2) *OC* contents (for details concerning soil acronyms see Section 3.1); the vertical dashed lines indicate the mean integration ranges. (**c**) Kendall’s rank correlations (*τ*) between the relative intensities of bands and *OC*, *H*, *N*, *S*, *K_d n_*, and *K_d ac_* values.

The obtained FT-IR spectra are presented in Figure 2 and Figure S4 in the Supplementary Materials. The band assignment was based on the literature data providing the FT-IR spectra of mineral species [44,45], functional groups of organic compounds [46], extracted fractions of SOM and enriched *HN* fractions [47,48], native soils with a significant content of organic matter [49], as well as on the results of the analysis of the correlations (Figure 2c) between the band intensities and the *OC*, hydrogen (*H*), nitrogen (*N*) and sulfur (*S*) contents determined in the HF-altered soils. Four bands in the spectra were assigned to the inorganic soil components: I-3700 (peak at 3700 cm^−1^, vibrations of kaolinite), II-3625 (smectite, illite), IX-1085 (quartz) and X-1035 (smectite, kaolinite and/or illite) [44,45,49]. Seven bands were attributed to SOM: III-3420 (O–H stretching of phenols and alcohols), IV-2920 (asymmetric stretching of aliphatic –CH_2_–), V-2850 (symmetric stretching of aliphatic –CH_2_–), VI-1725 (C=O stretching—mainly of carboxyl groups), VII-1640 (–HC=CH– stretching of aromatic rings, C=O stretching of amide, and N–H bending of amide and amine groups), VIII-1535 (–HC=CH– stretching of aromatic rings), and XI-795 (C–H out-of-plane bending of heteroaromatic and polynuclear aromatic compounds) [46,47,48,49]. More details can be found in Table S5 in the Supplementary Materials.

The obtained *K_d_* values from the experiments at the acidic (2.5–3.7) and near neutral (6.1–7.4) pH values were in the range of 16.9–437.4 and 3.4–135.5 mL/g, respectively. The organic carbon normalized adsorption coefficients (*K_OC_*; *K_OC_* = 100∙*K_d_*/*OC*) were in the range of 1125.0–2709.5 and 163.0–1051.2 mL/g. The comparison of the obtained *K_OC_* values with the *K_FOC_* values presented in Figure 1c (see the data for large *OC*) indicates that the sorption properties of SOM in the HF-altered soils did not change significantly.

The strongest correlation for the adsorption of TB in soils with the acidic pH (*K_d ac_* in Figure 2c) was obtained with the band VI-1725. At a pH of 2.5–3.7, 95.6–99.7% of TB existed in the protonated form. The highest correlation coefficients with *K_d n_* (soils with pH of 6.1–7.4) were obtained for the bands IV-2920 and V-2850. In this pH range between 92.2–99.6% of TB existed in the neutral form. None of the bands assigned to the inorganic components was found to be positively correlated with *K_d ac_* or *K_d n_*, suggesting that the adsorption of TB occurred predominantly on SOM in the HF-altered soils.

The variables attributed to SOM vibrations were analysed using PLSR and the *K_d_* values determined from experiments with acidic and-near neutral pH were examined together (n = 52), resulting in the following model (R^2^ = 0.958, LV = 2):

(15) $$K_{d}=-10.43+35.45_{\left(0.38,\;\;0.70\right)}\cdot IV-2920_{\Phi_{n}}+96.72_{\left(1.11,\;1.23\right)}\cdot VI-1725_{\Phi_{cat}}$$

The values given in brackets are the values of the standardized regression coefficient and VIP. According to Equation (15), the adsorption of the neutral form of TB was associated with the asymmetrical C–H stretching of the aliphatic groups, and that of the protonated form with the C=O vibrations (mainly carboxylic groups). However, replacing the *IV-*2920*_Φn_* variable with the *V*-2850*_Φn_* variable in Equation (15) led to a similar result (R^2^ = 0.953).

As mentioned earlier, Mosquera-Vivas et al. observed the dependence of TB adsorption on the amount of the aryl carbon and carbonyl carbon in soils [2]. However, the observations were made just on comparing the sequence of adsorption values in six soils and the sequences based on the contents of the aromatic rings and carbonyl C=O groups determined with the ^13^C nuclear magnetic resonance. Singh studied the adsorption mechanisms of hexaconazole, penconazole and triadimefon in the *HA* fraction using FT-IR spectroscopy and observed an increase in the intensity of peak in the region corresponding to the –CH=CH– vibrations of aromatic rings [21]. The author also observed a slight shift of C=O stretching vibrations in the *HA*-fungicide complexes, indicating that the carboxyl groups of *HA* interacted with the fungicide molecules. Thus, the interaction of TB cations with the carboxyl groups of SOM indicated in Equation (15) is consistent with the results of the above studies, but the TB molecule interactions with the aliphatic chains of SOM were not mentioned by the authors. Singh did not record FT-IR spectra at frequencies > 2000 cm^−1^ [21]. However, Gámiz et al. indicated that the *K_d_* values determined for the adsorption of TB at near-neutral pH in soil amended with oleate-intercalated hydrocalcite were 45 times higher when the content of the amended clay was increased by 1% [11]. The addition of the *VIII-1535_Φcat_* variable to Equation (15) increased the prediction of the *K_d_* variance by 0.07% only (insignificantly). However, this could be also related to the very low intensity of the band in our study (Figure S4 in the Supplementary Materials) and possible large errors in the estimation. Thus, the results of PLSR do not exclude existence of interactions between the aromatic rings of SOM and the benzene ring of TB but clearly indicate that the adsorption of TB molecules associated with the aliphatic chains of SOM was predominant. It is worth being mentioned that Singh also observed minor changes in the intensity of bands attributed to C–O stretching and O–H bending, which suggests the existence of hydrogen bonds between the pesticides and the hydroxyl groups of *HA* [21]. In our study, the vibrations associated with inorganic soil components were dominant in this region (IX-1085 in Figure 2b). 

### 2.4. Mechanisms of TB Adsorption on SOM and Clay Minerals

**Figure 3:**
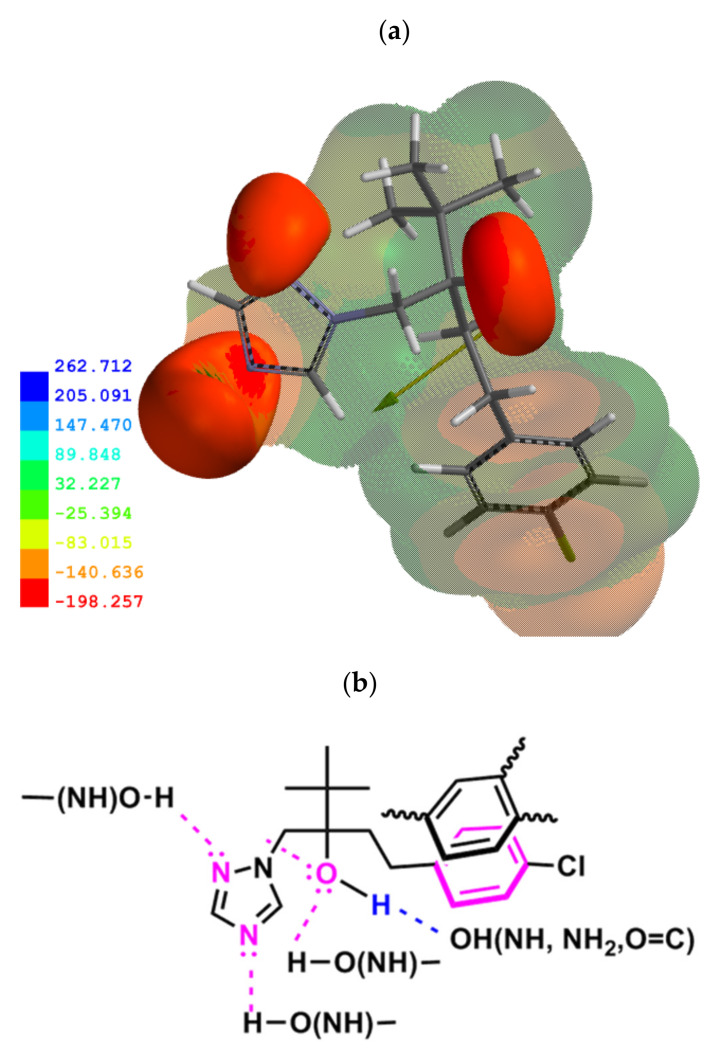
(**a**) Molecular electrostatic potential density (the superimposed electrostatic potential profile at −83.7 kJ/mol) and the dipole moment of TB; the deepest blue color—the most positive potential, the deepest red green—the most negative potential, the intermediate shades—the intermediate potential regions. (**b**) The proposed network of hydrogen bonds and π–π interactions between TB and SOM.

The molecular potential density distribution showed that the most negative potentials of TB molecule are located on the oxygen and N(4) and N(2) nitrogen atoms and a positive potential on the hydroxyl hydrogen (Figure 3a). The Mulliken charge distribution in the molecule also gave similar results (Figure S5 in the Supplementary Materials). This suggests that the TB molecule can form hydrogen bonds of different characteristics with the involvement of the hydroxyl group and that of the nitrogen atoms as acceptors of hydrogen. Moreover, there is also the possibility of π–π interactions of aromatic rings. The existence of such interactions was also indicated in previous studies on TB docking to the active sites of the CYP51 enzyme and the human serum albumin [50,51]. It seems that the formation of hydrogen bonds can be the dominant type of interaction of TB molecule in more polar regions of SOM with numerous functional groups (–OH, =C=O, =NH, or –NH_2_). In these regions, the TB molecule can even form a network of relatively stable directional hydrogen bonds acting as a multidentate ligand (Figure 3b).

The highest affinity of the *HN* fraction to the molecular form of TB (Equations (11) and (12)) is consistent with the finding that the adsorption of this TB form is associated with the symmetrical and asymmetrical C–H stretching of aliphatic chains. This is due to the diffusion of this hydrophobic adsorbate to the hydrophobic regions of *HN* [26] and its adsorption through hydrophobic interactions and weak van der Waals forces. Some segments of SOM contain hydrophobic microspaces [26,27] which are formed, e.g., by long alkyl linkers of *HN* and the residues of alkyl amino acids, especially the branched ones. Such hydrophobic spaces may be occupied by the hydrophobic regions of the TB molecule associated with the *tert*-butyl substituent. The possibility of this type of interactions was confirmed in the above-mentioned studies on TB docking to CYP51 and human serum albumin [50,51], and was suggested by many other authors [16,17,26,27].

To explain the influence of pH on adsorption, the pH-dependent changes in the structure of both adsorbate and adsorbent should be taken into account. The modeling results indicated protonation occurs on the N(4) atom of the triazole ring (Figure 3). This was signalized earlier by Mal’tseva et al. [16]. SOM contains two major classes of functional groups contributing to the cation exchange capacity and acidity of soils: carboxylic-type with a *pK_a_* of ~3–5, and phenolic-type with a *pK_a_* of ~8–11 [48,52]. Taking the above into account, it can be concluded that in soils with pH < 5, in which the protonated form of TB is dominant and a part of the carboxyl groups are dissociated, electrostatic attractions between the oppositely charged ions are dominant. Weber and Weber et al. suggested that ion exchange can be the principal adsorption mechanism of the protonated form of many s-triazines on SOM and clay minerals [53,54]. This finding was later confirmed by analytical methods [21,55].

*PA* was found as the second most significant variable responsible for the adsorption of the protonated TB form (Table 1), and more precisely, the hydrogen cations adsorbed in the exchangeable and permanent sorption sites. Determination of *PA* with the BaCl_2_-TEA solution at pH 8.2 suggested that a part of hydrogen was desorbed from the phenolic groups of SOM with a *pK_a_* < 10 and the carboxylic groups with a *pK_a_* > 6, and from sorption sites of clay minerals capable of desorption at this pH [42,48,52]. At pH 5 ± 2, in which the protonated form of TB can exist, dissociation of hydrogen cations from these groups is negligible. Therefore, it can be expected that direct protonation or complexation of the most basic N(4) atom of TB also occurred in these sorption sites:

(OM, Clay)^−^H^+^ + TB **⇌** (OM, Clay)^−^HTB^+^ **⇌** (OM, Clay)^−^ + HTB^+^ (16)

(OM, Clay)–H + TB **⇌** (OM, Clay)–H···TB (17)

Depending on the acidity of the functional groups of the adsorbent, the ionic structure was formed (Equation (16)) or a complex stabilized by the hydrogen bond (Equation (17)). The mechanism expressed in Equation (17) is only a slight modification of that (Equation (16)) proposed by Weber and Weber et al. [53,54]. Considering that TB is a multidentate ligand, in addition to the above-described complexation and ion exchange mechanisms, at low pH TB is able to form hydrogen bonds with its hydroxyl group and with the N(2) atom of the triazole ring, as well as to form π–π interactions with its benzene ring (Figure 3b).

## 3. Materials and Methods

### 3.1. Soils

The examined soil profiles, located in Southeastern Poland (Figure S1 in the Supplementary Materials), were selected from the Database of Polish Arable Mineral Soils from the Institute of Agrophysics of the Polish Academy of Science in Lublin [24]. Two of the selected soil profiles, cataloged in the Database as profiles 611 and 805, were classified as Arenosols [22], three (profiles 564, 590 and 824) as Luvisols, and one (profile 587) as Chernozem. The soil sampling and sample preparation procedures were described elsewhere [8]. The physical–chemical properties of the air-dried and sieved (2 mm sieve) soil samples are shown in Table S1 in the Supplementary Materials. The first letter in the name of the soil samples (e.g., A611Ap or C587AC) is an abbreviation of their soil group (A–Arenosol, L–Luvisol, C–Chernozem), the three-digit number is the soil profile number in the Database [24], and the letters at the end represent the soil horizon.

The soil texture (*Sand*, *Silt*, and *Clay* (%)) was determined using the pipette method [56]. The organic complexes and poorly crystallized oxides of Al, Fe, and Mn (denoted as *Al*(*T*), *Fe*(*T*), and *Mn*(*T*) (g/kg)) as well as exchangeable, occluded on Fe and Mn oxides, and complexed in SOM Cu (*Cu*(*T*) (mg/kg)) [33] were dissolved in the dark with Tamm’s reagent (0.175 M ammonium oxalate–0.1 M oxalic acid; 12 h, 25 °C, 1:35 (g/mL) soil to solution ratio [57]) and analyzed using a Varian AA280FS Atomic Absorption Spectrometer.

The soil organic carbon (*OC* (%)) was determined using a SSM-5000A solid sample module of a Shimadzu TOC-VCSH analyzer. Corrections for inorganic carbon were done for samples containing carbonates. Black carbon (*BC* (%)) was determined using altered soil samples (fumigated for 24 h in the vapor of concentrated HCl to remove carbonates [58], and then heated in a muffle furnace with air access at 375 °C for 24 h to oxidize SOM [59]) and the same solid sample module of the TOC analyzer. The fractions of soil humic substances were determined by extraction methods with 0.1 M sodium hydroxide (SH) [60] and 0.1 M sodium pyrophosphate (SP) [61]. The humins (*HN*) content was estimated assuming that *HN*(*SH*) = *OC* *− FA*(*SH*) *− HA*(*SH*) and *HN*(*SP*) = *OC − FA*(*SP*) *− HA*(*SP*) [37].

The effective cation exchange capacity (*ECEC* (cmol(+)/kg)) was determined using a procedure based on a solution of 0.0025 M BaCl_2_ [62], while the exchangeable acidity (*EA* (cmol(+)/kg)) was calculated by potentiometric titration (0.005 M NaOH, to pH 7.8) of the supernatants with BaCl_2_ [63]. The same supernatants were used for calculating the content of exchangeable Al (*Al*(*EA*) (cmol(+)/kg) [63]) after its determination with a Varian Carry 60 UV-Vis Spectrophotometer (λ = 550 nm, eriochrome cyanine R, pH 5.5 [64]). The potential cation exchange capacity (*PCEC* (cmol(+)/kg)) was determined with a solution of 0.5 M BaCl_2_ and 0.17 M triethanolamine (BaCl_2_-TEA), the pH of which was adjusted to 8.2 with HCl [42,65]. The supernatants with BaCl_2_-TEA were used for potentiometric titration (0.1 M HCl, to pH 5.2) to calculate potential acidity (*PA* (cmol(+)/kg)) [42]. The contents of extractable Al in the soils (*Al*(*PA*) (cmol(+)/kg)) were estimated by spectrophotometric determination of Al in the supernatants.

More information concerning the above analyses is presented in the Supplementary Materials. The procedures for the determination of specific surface area based on N_2_ and H_2_O vapor adsorption isotherms (*SSA*(*aN*_2_) and *SSA*(*a*H_2_O) (m^2^/g)) as well as that of the mean pore radius from the desorption isotherms (*r*(*d*N_2_)*mean* and *r*(*d*H_2_O)*mean* (nm)) were described elsewhere [8].

### 3.2. Sample Preparation and FT-IR Analysis

Thirteen soils with the largest SOM contents were prepared for FT-IR spectroscopy measurements. A modified procedure by Rumpel et al. was used [43]. The samples were weighed into polypropylene tubes, agitated in a rotator (24 h, 1:2.5 ratio (g/mL) of soil to 2% HF solution), centrifuged (15 min, 3000 *g*, 20 °C), and the supernatants were discarded. Agitation was repeated three times with new portions of 2% HF, and four times with redistilled water. Next, the samples were freeze-dried to remove water. The contents (%) of *OC*, nitrogen (*N*), sulphur (*S*), and hydrogen (*H*) in the altered soils were determined using a Vario El cube CHNS elemental analyzer.

One milligram of samples was mixed with 400 mg KBr and ground with an agate mortar, and the pellets were pressed. The FT-IR spectra were recorded in the range of 4000–700 cm^−^^1^, with a resolution of 0.964 cm^−^^1^ and 32 scans per sample using a Bruker Vertex 70 FT-IR spectrometer. The obtained spectra were corrected for baseline and integrated with OriginPro 2015 software. The band intensities obtained after integration were adjusted to the SOM content, assuming that vibration intensities with peaks at 2920 and 1725 cm^−^^1^ corresponded to the internal standards of organic carbon. The elemental composition of the samples, details of the integration methods, and intensities of the integrated spectra bands are presented in the Supplementary Materials.

### 3.3. Batch Adsorption Experiments

A stock methanol solution of TB (500 mg/L) and aquatic TB solutions (0.5, 1.0, 1.5, 2.0, 2.5, and 5.0 mg/L in 0.01 M CaCl_2_) were prepared using a certified analytical standard (99.9 ± 0.1%; Institute of Organic Industrial Chemistry, Warsaw, Poland) with HPLC (high-performance liquid chromatography)-grade methanol or sterile redistilled water. All the other solvents and chemicals used were of analytical or HPLC grade.

The batch adsorption experiments were carried out according to the OECD Guideline 106 [25] at 20 ± 0.5 °C. The soil/solution ratio used was 1:10. For the determination of the adsorption isotherms, triplicate soil samples (1 g) were weighed to glass tubes and 0.01 M CaCl_2_ containing 5 × 10^−^^5^ M HgCl_2_ (biocide; 2 mL) was added. After 12 h of equilibration the respective TB solutions were added (8 mL; initial concentrations: 0.4–2.0 μg/mL), and the tubes were agitated with an overhead shaker (96 h). Our previous study [8] indicated that 96 h of agitation is necessary to reach adsorption equilibrium or near-equilibrium. Then, pH was measured in samples with the largest initial TB concentration with a glass electrode. The tubes were centrifuged (10 min, 3300 *g*, 20 °C), and the liquid phase was sampled for HPLC analysis.

The *K_d_* values were determined in HF-altered soils using quadruple soil samples (50 mg) to which 0.012 M CaCl_2_ containing 3·10^−^^5^ M HgCl_2_ (1.0 mL) was added. In duplicate samples, pH was adjusted to ~3 and to ~7 within 12 h by using HCl or NaOH. Then, redistilled water was added to obtain the total solution volume of 1.2 mL, and 5.0 μg/mL TB was added (1.8 mL; initial concentration: 3.0 μg/mL). The tubes were agitated (96 h), and pH was measured. After centrifugation (10 min, 3300 *g*, 20 °C), the liquid phase was sampled for HPLC analysis.

HPLC measurements were carried out as described in our previous study [8]. Adsorption of TB was determined from the difference between its initial and equilibrium concentrations [25].

### 3.4. Molecular Modeling and Statistical Analysis

The model of a TB molecule was built with a standard bond length and angles using PC SPARATN’10 Pro Ver. 1.1.0 molecular modeling software [66]. Energy was minimized by molecular mechanical methods, and the conformer of the lowest energy was taken for further studies. The structure of TB was optimized by the Hartree–Fock method at 6-311G** level and equilibrium geometry at the ground state [66]. Ab initio 6-311+G** basis set is a valence triple-zeta polarized basis set that adds a set of polarizing d-functions on heavy atoms and a set of polarization p-functions on hydrogen (6-311G(d,p) [67]). The charge of atoms from the Mulliken charge was determined as described by Singh and Kollman [68].

The PLSR analysis was carried out with XLSTAT 2018.7 software [69], while other statistical analyses were performed with Statgraphics Centurion XVII (Statpoint Technologies, Inc., Warrenton, VA, USA).

## 4. Conclusions

This study indicated that TB adsorption was the classical process of adsorption of an organic base which is inversely correlated with soil pH. The neutral form of TB was found to be bound mainly in the *HN* fraction, most likely through the formation of hydrogen bonds, and through hydrophobic and π–π interactions. Adsorption of this form of TB was strongly correlated with the C–H stretching of the aliphatic groups of SOM.

At pH < 5, the protonated form of TB, obtained by protonation of the triazole N(4) atom, was dominant. This form of TB was also adsorbed mainly in the *HN* fraction, but its adsorption was larger in the presence of hydrogen cations adsorbed in the permanent and exchangeable sorption sites of the *FA* fraction and clay minerals. Adsorption of protonated TB was strongly correlated with the C=O stretching vibrations of SOM, which suggests the dominant contribution of the carboxyl group in the adsorption, and ion exchange as the dominant mechanism of adsorption. Apart from ion exchange, a complexation reaction also likely occurred between the N(4) atom of TB and hydrogen in the permanent sorption sites of SOM and clay minerals. The greatest affinity of *HN* toward the TB cations is surprising. This may indicate that not only the N(4) atom but also the hydroxyl group and N(2) atom are involved in the adsorption of this form of TB, and the fungicide behaves in soils as a multidentate ligand.

Thus, our study has clearly shown that in the case of TB, and probably also of other organic bases, the *HN* fraction is the crucial fraction affecting adsorption. Despite the fact that in many soils it is the dominant fraction, so far it has been the least investigated fraction of SOM. Soil pH was another soil property which had a decisive influence on TB adsorption. It can be hypothesized that liming or using fertilizers that increase the soil pH may cause desorption of the part of TB accumulated in the top soil layer and its movement down the soil profile.

It can be also concluded that the pH-dependent processes of TB adsorption in soils can be easily and precisely described assuming that the SOM and *PA* affect its adsorption significantly. The model assuming that both forms of an organic base are adsorbed on SOM, and adsorption of the protonated form is affected additionally by the second variable–soil property, can be easily implemented into the pH-dependent modules of the pesticide leaching programs. It can be hypothesized that the model should properly describe adsorption of a large part of basic pesticides.

## Figures and Tables

**Table 1:** This relationship between *K_d_* (mL/g) and soil properties according to the PLSR analysis.

No.	Equation Standardized Regression Coefficient; VIP	R^2^/LV ^a^
(4)	*K_d_* = 1.69 + 7.49∙(*OC-BC*)*_Φn_*+ 4.84∙*BC_Φn_* + 20.52∙(*OC-BC*)*_Φcat_*+ 51.23∙*BC_Φcat_* 0.56; 1.08* * 0.10; 0.63 0.74; 1.26* * 0.08; 0.91	0.903/3
(5)	*K_d_* = 1.74 + 8.79∙(*OC-BC*)*_Φn_*+ 20.89∙(*OC-BC*)*_Φcat_* 0.66; 0.93 0.76; 1.07	0.902/1
(6)	*K_d_* = 1.24 + 27.64∙*FA*_(*SP*)*Φn*_ + 5.44∙*HA*_(*SP*)*Φn*_ + 3.96∙*HN*_(*SP*)*Φn*_ + 36.78∙*FA*_(*SP*)*Φcat*_ + 43.68∙*HA*_(*SP*)*Φcat*_ + 12.75∙*HN*_(*SP*)*Φcat*_ 0.24; 0.94 0.14; 0.55 0.26; 1.03 0.25; 1.00 0.28; 1.11 0.31; 1.23	0.867/1
(7)	*K_d_* = 1.67 + 10.86∙*HN*_(*SP*)*Φn*_ + 47.43∙*FA*_(*SP*)*Φcat*_ + 19.08∙*HN*_(*SP*)*Φcat*_ 0.70; 1.01 0.32; 0.90 0.46; 1.08	0.931/2
(8)	*K_d_* = 1.68 + 13.20∙*FA*_(*SH*)*Φn*_ + 10.25∙*HA*_(*SH*)*Φn*_ + 6.72∙*HN*_(*SH*)*Φn*_ + 34.46∙*FA*_(*SH*)*Φcat*_ + 24.58∙*HA*_(*SH*)*Φcat*_ + 13.40∙*HN*_(*SH*)*Φcat*_ 0.11; 0.94 0.07; 1.03 0.59; 1.03 0.27; 0.94 0.21; 1.01 0.28; 1.04	0.894/3
(9)	*K_d_* = 1.74 + 6.86∙*HN*_(*SH*)*Φn*_ + 41.68∙*HN*_(*SH*)*Φcat*_ 0.60; 0.80 0.88; 1.17	0.895/1
(10)	*K_d_* = 0.39 + 10.08∙(*OC-BC*)*_Φn_*+ 18.60∙(*OC-BC*)*_Φcat_* + 0.54∙*PA_Φcat_* 0.76; 1.11 0.67; 1.25 0.21; 0.46	0.955/2
(11)	*K_d_* = 0.38 + 12.16∙*HN*_(*SP*)*Φn*_ + 25.32∙*HN*_(*SP*)*Φcat*_ + 0.85∙*PA_Φcat_* 0.79; 1.08 0.61; 1.28 0.34; 0.46	0.982/3
(12)	*K_d_* = 0.01 + 35.45∙*HA*_(*SH*)*Φn*_ + 8.33∙*HN*_(*SH*)*Φn*_ + 24.48∙*HN*_(*SH*)*Φcat*_ + 0.84∙*PA_Φcat_* 0.25; 1.18 0.73; 0.97 0.51; 1.18 0.33; 0.52	0.956/3

^a^ LV—number of latent vectors.

## Data Availability

All relevant data are within the text and the Supplementary Materials.

## Supplementary Materials

The following are available online, Table S1: Physico-chemical properties of soils, Table S2: Results of fitting of the Linear and Freundlich models to the adsorption data presented in Figure S2, Table S3: The Pearson’s (bottom-left) and Kendall’s correlation coefficients and *p-*values for *K_d_* (18 soils, triplicate samples, n = 54) and the soil properties from Table S1, Table S4: Elemental composition, corrected band intensities, *K_d ac_*, *K_d n_*, and pH values from the adsorption experiments in the HF-altered soils, Table S5: Band assignments of the FT-IR spectra of HF-altered soils, Figure S1: Location of the examined soil profiles on the map of Poland, Figure S2: Adsorption isotherms in soils from the six examined profiles, Figure S3: Contents of *FA*, *HA* and *HN* determined based on the extractions with (a) 0.1 M Na_4_P_2_O_7_ and (b) 0.1 M NaOH, Figure S4: Selected ranges of FT-IR spectra of 13 HF-altered soils, shifted vertically to avoid overlapping and arranged in order from the largest (A611Ap) to the smallest (L590Bt1) *OC*, Figure S5: (a) The Milliken charge distribution for molecular and (b) protonated at N(4) forms of TB.
