# Supplementary material for: Mechanisms of Tebuconazole Adsorption in Profiles of Mineral Soils
Source: Molecules. 2021 Aug 4;26(16):4728. doi: 10.3390/molecules26164728 (PMC8398351; doi:10.3390/molecules26164728)
Supplement: Supplementary file 1 [file molecules-26-04728-s001.zip › molecules-1277719-supplementary.pdf]

Supplementary Materials for:

Mechanisms of tebuconazole adsorption in profiles of mineral soils

Marcin Siek[a], Tadeusz Paszko[a]*, Maria Jerzykiewicz[b], Joanna Matysiak[a], Urszula Wojcieszek[a]

[a]*Department of Chemistry, University of Life Sciences, Akademicka 13, 20-950 Lublin, Poland*

[b]*Faculty of Chemistry, University of Wrocław, F. Joliot-Curie 14, 50-383 Wrocław, Poland.*

**Corresponding author*:

E-mail address: tadeusz.paszko@up.lublin.pl

A. Soils

Table S1. Physico-chemical properties of soils.

Soil	A611Ap	A611BC	A611C	L590Ap	L590Bt1	L590Bt2	L564Ap	L564Bt1	L564Bt2	C587Ap	C587A2	C587AC	A805Ap	A805BC	A805C	L824Ap	L824Bt1	L824Bt2
(Latitude; Longitude)	(51°23’56’’N; 22°14’14’’E)			(51°26’17’’N; 22°10’14’’E)			(50°50’36’’N; 23°21’57’’E)			(50°27’36’’N; 23°47’35’’E)			(50°51’18’’N; 20°45’38’’E)			(50°52’51’’N; 20°45’48’’E)		
	Olempin			Dęba			Skierbieszów			Ulhówek			Górno			Leszczyny		
Sand (%)[a]	88.5	96.5	98.0	78.0	57.0	48.0	18.0	16.0	14.0	16.5	15.5	14.5	85.0	95.0	89.5	51.5	62.5	66.5
Silt (%)	9.7	2.4	1.0	17.7	20.0	22.0	72.2	71.5	61.1	70.6	64.6	67.7	12.3	4.1	7.2	39.8	22.1	22.5
Clay (%)	1.8	1.1	1.0	4.3	23.0	30.0	9.8	12.5	24.9	12.9	19.9	17.8	2.7	0.9	3.3	8.7	15.4	11.0
pH[b]	4.96	4.62	4.54	4.45	4.75	4.58	6.97	6.78	6.85	7.08	7.32	7.48	4.20	5.06	5.57	5.46	4.52	4.57
Al(T) (g/kg)[c]	0.51	0.39	0.31	0.58	0.84	0.81	0.68	0.77	1.22	0.88	1.25	0.92	0.40	0.17	0.23	0.85	0.71	0.62
Fe(T) (g/kg)	1.43	0.49	0.40	2.44	5.78	4.81	3.53	3.57	4.35	2.58	3.83	3.71	2.15	0.38	0.94	4.56	3.86	5.53
Mn(T) (g/kg)	0.08	0.01	0.02	0.21	0.24	0.16	0.23	0.24	0.19	0.19	0.20	0.16	0.09	0.01	0.01	1.02	0.06	0.34
Cu(T) (mg/kg)	0.68	0.33	0.10	1.60	1.96	3.41	3.70	3.23	3.19	5.40	4.24	3.36	1.16	0.20	0.43	3.56	1.80	2.11
OC (%)[d]	1.130	0.090	0.034	0.882	0.159	0.088	1.355	0.478	0.313	1.921	1.314	0.666	0.681	0.042	0.041	1.075	0.190	0.109
BC (%)[e]	0.000	0.000	0.000	0.016	0.001	0.000	0.091	0.047	0.024	0.434	0.343	0.213	0.020	0.005	0.009	0.133	0.017	0.000
HA(SP) (%)[f]	0.176	0.018	0.001	0.171	0.032	0.029	0.151	0.111	0.027	0.397	0.566	0.209	0.111	0.015	0.016	0.140	0.044	0.021
FA(SP) (%)	0.164	0.033	0.026	0.180	0.055	0.030	0.155	0.070	0.060	0.149	0.119	0.108	0.138	0.024	0.025	0.177	0.069	0.049
HN(SP) (%)	0.790	0.039	0.007	0.531	0.072	0.029	1.049	0.297	0.226	1.375	0.629	0.349	0.432	0.003	0.001	0.758	0.077	0.039
HA(SH) (%)[g]	0.279	0.010	0.000	0.183	0.000	0.000	0.074	0.006	0.000	0.000	0.009	0.000	0.141	0.000	0.000	0.159	0.002	0.001
FA(SH) (%)	0.191	0.026	0.018	0.201	0.042	0.021	0.161	0.064	0.045	0.054	0.071	0.013	0.156	0.020	0.016	0.213	0.060	0.044
HN(SH) (%)	0.660	0.054	0.016	0.498	0.117	0.067	1.120	0.409	0.268	1.867	1.233	0.653	0.383	0.023	0.025	0.703	0.128	0.065
ECEC (cmol(+)/kg)[h]	1.528	0.760	0.784	2.155	8.469	10.825	9.587	8.215	12.212	12.429	12.196	11.735	1.346	0.746	1.531	5.394	5.919	4.677
EA (cmol(+)/kg)[i]	0.124	0.216	0.256	0.356	0.182	0.225	0.005	0.000	0.000	0.005	0.000	0.000	0.469	0.070	0.004	0.054	0.477	0.287

Al(EA) (cmol(+)/kg)[j]	0.036	0.161	0.191	0.107	0.044	0.075	0.000	0.000	0.000	0.000	0.000	0.000	0.211	0.006	0.000	0.000	0.228	0.139
PCEC (cmol(+)/kg)[k]	4.763	1.252	1.144	5.628	11.359	12.660	12.249	10.269	15.597	16.128	15.799	14.370	3.715	0.699	1.794	9.784	9.266	7.218
PA (cmol(+)/kg)[l]	5.891	2.388	1.921	6.048	7.765	9.048	3.295	3.387	5.011	2.696	2.887	1.419	5.795	1.417	1.730	9.229	7.865	6.419
Al(PA) (cmol(+)/kg)[j]	0.698	0.406	0.295	0.558	0.158	0.135	0.168	0.048	0.019	0.035	0.058	0.025	0.619	0.192	0.161	0.396	0.357	0.338
SSA(aN2) (m^2/g)[m]	0.40	1.28	1.34	2.32	20.04	27.86	6.89	11.25	29.61	14.05	17.05	17.87	1.02	0.77	2.64	7.34	14.48	12.54
SSA(aH2O) (m^2/g)	7.84	2.43	1.85	11.03	38.90	54.61	28.16	25.38	51.78	46.15	47.88	41.12	6.03	1.19	5.07	21.63	29.00	22.24
r(dN2)mean (nm)	7.04	4.34	3.78	4.92	3.42	3.34	3.28	3.15	2.86	3.03	2.89	3.36	6.15	4.01	3.61	4.41	3.24	3.45
r(dH2O)mean (nm)	4.43	4.78	4.75	4.73	4.22	4.10	4.32	4.13	3.88	4.09	3.88	4.08	4.90	5.60	4.95	4.83	4.25	4.25

[a] determined using the pipette method [1]; [b] determined in 0.01 M $CaCl_2$; [c] extracted with Tamm's reagent [2] and analyzed using a Varian AA280FS Atomic Absorption Spectrometer; [d] determined using a SSM-5000A solid sample module of Shimadzu TCSH analyzer; [e] the samples were fumigated for 24 h in the vapor of concentrated HCl [3], heated in a muffle furnace with air access at 375°C for 24 h [4], and analyzed using a SSM-5000A solid sample module of Shimadzu TCSH analyzer; [f] determined after extraction with 0.1 M sodium pyrophosphate [5]; [g] determined after extraction with 0.1 M sodium hydroxide [6]; [h] extracted with 0.0025 M $BaCl_2$ [7]; [i] determined by the potentiometric titration with 0.005 M NaOH to pH of 7.8 [8]; [j] determined with a Varian Carry 60 UV-Vis Spectrophotometer – λ = 550 nm, eriochrome cyanine R, pH 5.5 [9]; [k] determined after extraction with a solution of 0.5 M $BaCl_2$ and 0.17 M triethanolamine ($BaCl_2$-TEA), pH 8.2 [10, 11]; [l] determined by the potentiometric titration with 0.1 M HCl to pH of 5.2 [11]; [m] details were described elsewhere [12].

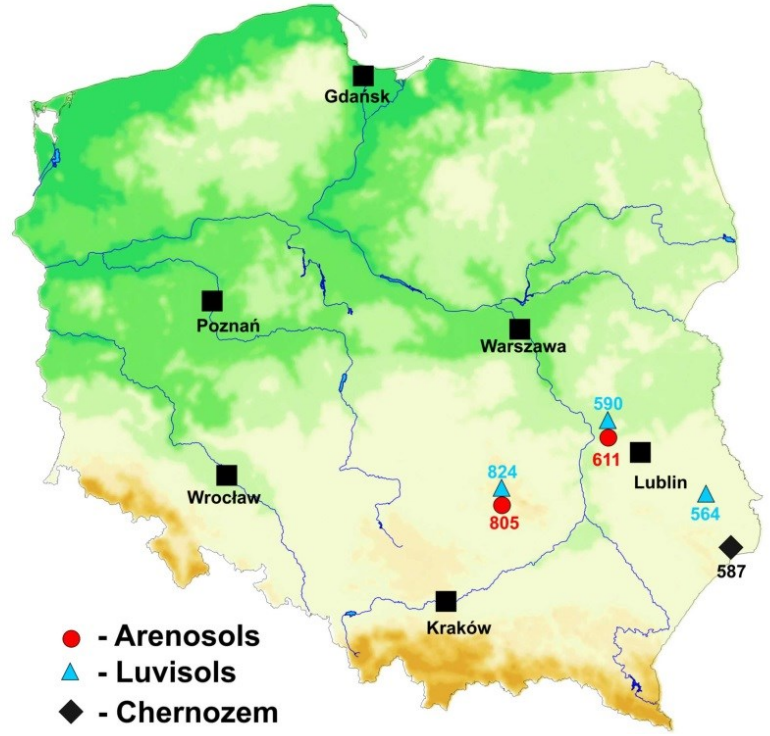

Figure S1. Location of the examined soil profiles on the map of Poland.

Fractionation of organic matter

Two sequential extraction methods were selected. The first method was based on extraction with 0.1 M sodium pyrophosphate (SP) at pH 10, saturated with N_2 [5]. The fumigated for 24 h in the concentrated HCl duplicate 3 g soil samples were shaken in a Biosan Multi RS-60 rotator for 24 h with 25 mL of the 0.1 M $Na_4P_2O_7$ solution. Each solution was separated after centrifugation (30 min., 3000 g, 20°C) and part of the extract was used for the organic carbon (*OC*) determination with a SSM-5000A solid sample module of a Shimadzu TOC-VCSH analyzer. For each soil the sum of humic and fulvic acids was calculated (*HA(SP)+FA(SP)* (%)). The 9 mL portions of each extract were also acidified with 6 M HCl to pH 1, and after 16 h the precipitated HAs were centrifuged (30 min., 3500 g, 20°C). In the collected supernatants *OC* was determined, next the FA contents (*FA(SP)* (%)) were calculated. The fraction of humins insoluble in the 0.1 M $Na_4P_2O_7$ (*HN(SP)* (%)) was calculated from the difference: $HN(SP) = OC - HA(SP)+FA(SP)$ [13].

Moreover, the simplified method of sequential extraction based on 0.1 M NaOH (SH) was used [6]. Briefly, 20 mL of 0.1 M NaOH saturated with N_2 was added to the duplicate 3 g soil samples fumigated for 24 h in the concentrated HCl. The suspensions were agitated for 4 h on the rotator, left for sedimentation for 12 h, centrifuged (30 min., 3000 g, 20°C), and the extract solutions were collected. Part of each solution was used for

organic carbon determination in order to calculate the sum of HA and FA in the soils (*HA(SH)+FA(SH)*). The 10 mL samples of each extract were acidified to pH 1 with 6 M HCl, left overnight to precipitate HA, centrifuged (30 min., 3500 g, 20°C), and in the collected supernatants organic carbon was determined. The *HA(SH)*, *FA(SH)*, and *HN(SH)* contents (%) were calculated in the same way as for the method with sodium pyrophosphate.

Cation exchange capacity and exchangeable/extractable acidity determination

The effective cation exchange capacity (*ECEC* (cmol(+)/kg) was determined using a method with 0.0025 M solution of $BaCl_2$ [7]. Briefly, 16.5 mL volumes of 0.0025 M $BaCl_2$ solution were added three times to the duplicate 7.5 g samples of dry weight soil placed in 30 mL polypropylene tubes. Each time the samples were agitated on the rotator for 1 h, centrifuged (10 min., 3500 g, 20°C), and the supernatants were collected into 50 mL volumetric flasks. The fourth time, after adding 16.5 mL of 0.0025 M $BaCl_2$, the tubes were agitated for 12 h, centrifuged, and the supernatant was removed. Next, 26 mL of 0.02 M $MgSO_4$ was added to each tube and the tubes were agitated for 12 h, centrifuged, and supernatants were collected for Mg determination with the Varian AA280FS Atomic Absorption Spectrometer. On this basis the *ECEC* values were calculated [7]. The supernatants collected in the 50 mL flasks were filtrated, and duplicate 17 mL portions of filtrate were used for the potentiometric titration with 0.005 M NaOH to the endpoint pH of 7.8, and on this basis the exchangeable acidity (*EA* (cmol(+)/kg) was calculated [8]. The same filtrates were used for the Al determination (Varian Carry 60 UV-Vis Spectrophotometer, $\lambda = 550$ nm, eriochrome cyanine R, pH 5.5 [9]), and next for the exchangeable Al contents (*Al(EA)* (cmol(+)/kg) calculation [8].

The potential cation exchange capacity (*PCEC* (cmol(+)/kg) was determined using a method with the solution of 0.5 M $BaCl_2$ and 0.17 M triethanolamine ($BaCl_2$-TEA) acidified to pH 8.2 with HCl [10, 11]. The 16.5 mL volumes of $BaCl_2$-TEA were added three times to the duplicate 5.0 g samples of dry weight soil. The tubes were agitated on the rotator for 1, 2, and 12 h, centrifuged (10 min., 3500 g, 20°C), and the supernatants were collected into 50 mL volumetric flasks. The fourth time 26 mL of redistilled water was added, tubes were agitated for 0.5 h, centrifuged, and the supernatant was removed. Next, 26 mL of 0.02 M $MgSO_4$ was added to each tube and the tubes were agitated for 12 h, centrifuged, supernatants were collected for Mg determination with the Atomic Absorption Spectrometer, and the obtained data were used for PCEC calculation [10]. After

filtration of the supernatants collected in the 50 mL flasks, the duplicate 16 mL portions of filtrates (as well as the blank $BaCl_2$-TEA solutions) were used for the potentiometric titration with 0.1 M HCl to the endpoint pH of 5.2. Based on the difference in titration between the blank and supernatant solutions the potential (called also as extractable or titratable) acidity (*PA* (cmol(+)/kg) was calculated [11]. The contents of the extractable Al in the soils (*Al(PA)* (cmol(+)/kg) were determined based on the spectrophotometric determination of Al in the supernatants, similarly as for *Al(EA)*.

B. Adsorption experiments in native soils.

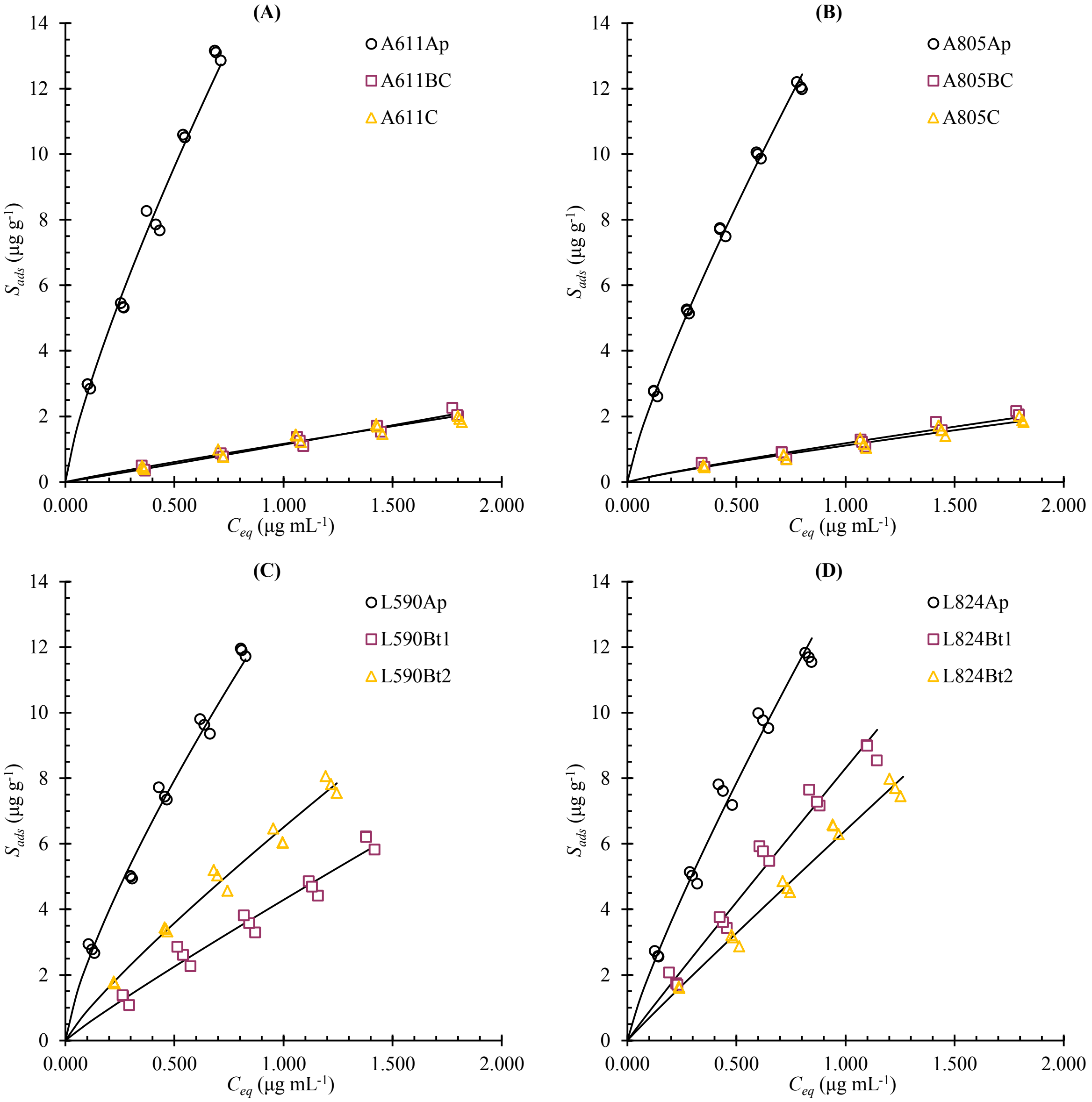

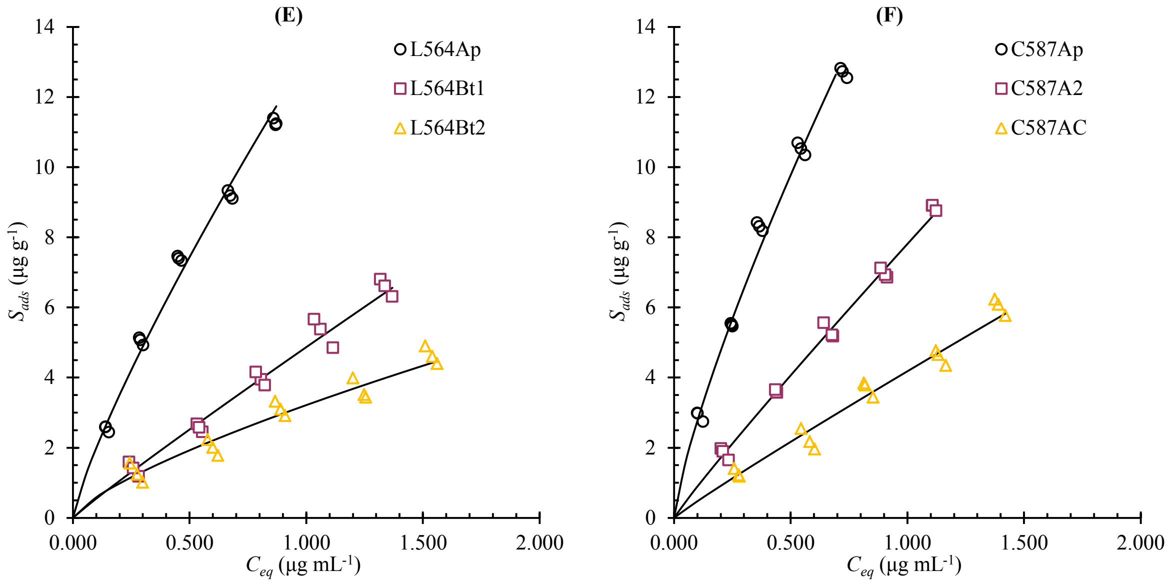

Figure S2. Adsorption isotherms in soils from the six examined profiles. Black lines – Freundlich model (parameters are provided in Table S2).

Table S2. Results of fitting the Linear and Freundlich models to the adsorption data presented in Figure S2.

	Linear model $S_{eq}^{ads} = K_d \cdot C_{eq}$			Freundlich model $S_{eq}^{ads} = K_F \cdot C_{eq}^{1/n}$				
Soil	K_d (mL/g)	R^2/R_a^2	*RSS* [a]	K_F ($\mu g^{1-1/n}$ $(mL)^{1/n}$ g^{-1})	*1/n*	R^2/R_a^2	*RSS*	Isotherm type [14]
A611Ap	19.20	0.973/0.971	5.29	16.65	0.79	0.984/0.981	3.51	L
A611BC	1.15	0.972/0.970	0.15	1.13	1.04	0.969/0.964	0.15	C
A611C	1.13	0.936/0.931	0.29	1.16	0.94	0.962/0.956	0.24	L
A805Ap	16.24	0.962/0.959	6.37	14.93	0.82	0.994/0.993	1.00	L
A805BC	1.15	0.963/0.960	0.18	1.20	0.88	0.957/0.952	0.20	C
A805C	1.07	0.957/0.954	0.18	1.11	0.86	0.959/0.952	0.17	C
L590Ap	15.20	0.956/953	6.79	13.44	0.76	0.980/0.977	2.31	L
L590Bt1	4.28	0.963/0.960	1.56	4.29	0.93	0.959/0.952	1.50	C
L590Bt2	6.54	0.962/0.959	2.54	6.50	0.86	0.979/0.976	1.40	L
L824Ap	15.08	0.947/0.942	8.46	14.15	0.85	0.977/0.973	3.64	L
L824Bt1	8.29	0.968/0.965	3.11	8.30	0.98	0.970/0.966	2.90	L
L824Bt2	6.44	0.981/980	1.38	6.41	0.97	0.982/0.979	1.35	C
L564Ap	13.93	0.933/0.928	9.41	13.14	0.82	0.986/0.984	2.19	L
L564Bt1	4.91	0.971/0.968	1.53	4.87	0.95	0.964/0.958	1.58	C
L564Bt2	3.12	0.897/0.889	2.25	3.21	0.74	0.936/0.925	1.39	L
C587Ap	19.00	0.923/0.917	14.11	16.85	0.79	0.984/0.981	2.93	L
C587A2	7.91	0.991/0.990	0.86	7.81	0.94	0.991/0.990	0.83	C
C587AC	4.22	0.965/0.962	1.52	4.18	0.95	0.962/0.956	1.65	C

[a] residual sum of squares.

Table S3. The Pearson's (bottom-left) and Kendall's (top-right) correlation coefficients and *p*-values for K_d (18 soils, triplicate samples, n = 54) and the soil properties from Table S1.

	K_d	pH	Sand	Silt	Clay	Al(T)	Fe(T)	Mn(T)	Cu(T)	OC	BC	FA(SP)	HA(SP)	HN(SP)	FA(SH)	HA(SH)	HN(SH)	ECEC	EA	Al(EA)	PCEC	PA	Al(PA)	SSA(aN_2)	SSA(aH_2O)	r(dN_2)mean	r(dH_2O)mean
K_d		-0.171	-0.238	0.374	0.101	0.277	0.192	0.416	0.463	0.821	0.343	0.850	0.768	0.836	0.810	0.644	0.766	0.241	0.210	-0.001	0.325	0.507	0.338	-0.095	0.250	0.141	-0.124
		0.212	*0.083*	*0.007*	*0.462*	*0.044*	*0.162*	*0.003*	*0.001*	*<0.001*	*0.013*	*<0.001*	*<0.001*	*<0.001*	*<0.001*	*<0.001*	*<0.001*	*0.080*	*0.127*	*0.994*	*0.018*	*<0.001*	*0.014*	*0.492*	*0.069*	*0.306*	*0.368*
pH	-0.067		-0.591	0.548	0.257	0.494	-0.025	0.125	0.492	0.343	0.634	0.079	0.345	0.288	-0.165	-0.283	0.439	0.544	-0.949	-0.891	0.540	-0.562	-0.726	0.295	0.349	-0.548	-0.455
	0.631		*<0.001*	*<0.001*	*0.061*	*<0.001*	*0.855*	*0.364*	*<0.001*	*0.013*	*<0.001*	*0.567*	*0.012*	*0.036*	*0.229*	*0.039*	*0.001*	*<0.001*	*<0.001*	*<0.001*	*<0.001*	*<0.001*	*<0.001*	*0.032*	*0.011*	*<0.001*	*0.001*
Sand	-0.143	-0.784		-0.907	-0.835	-0.922	-0.645	-0.589	-0.868	-0.540	-0.721	-0.375	-0.585	-0.496	-0.179	0.145	-0.651	-0.932	0.619	0.636	-0.940	-0.162	0.738	-0.785	-0.884	0.759	0.845
	0.302	*<0.001*		*<0.001*	*<0.001*	*<0.001*	*<0.001*	*<0.001*	*<0.001*	*<0.001*	*<0.001*	*0.006*	*<0.001*	*<0.001*	*0.194*	*0.292*	*<0.001*	*<0.001*	*<0.001*	*<0.001*	*<0.001*	*0.238*	*<0.001*	*<0.001*	*<0.001*	*<0.001*	*<0.001*
Silt	0.214	0.842	-0.971		0.655	0.777	0.562	0.697	0.905	0.655	0.760	0.488	0.626	0.622	0.323	0.019	0.732	0.835	-0.544	-0.622	0.849	0.156	-0.591	0.596	0.734	-0.697	-0.693
	0.120	*<0.001*	*<0.001*		*<0.001*	*<0.001*	*<0.001*	*<0.001*	*<0.001*	*<0.001*	*<0.001*	*<0.001*	*<0.001*	*<0.001*	*0.019*	*0.888*	*<0.001*	*<0.001*	*<0.001*	*<0.001*	*<0.001*	*0.257*	*<0.001*	*<0.001*	*<0.001*	*<0.001*	*<0.001*
Clay	-0.126	0.311	-0.701	0.511		0.858	0.839	0.457	0.717	0.216	0.362	0.092	0.344	0.172	-0.028	-0.340	0.340	0.893	-0.303	-0.308	0.874	0.404	-0.678	0.965	0.965	-0.748	-0.862
	0.366	*0.022*	*<0.001*	*<0.001*		*<0.001*	*<0.001*	*0.001*	*<0.001*	*0.116*	*0.008*	*0.504*	*0.012*	*0.210*	*0.839*	*0.013*	*0.013*	*<0.001*	*0.027*	*0.025*	*<0.001*	*0.003*	*<0.001*	*<0.001*	*<0.001*	*<0.001*	*<0.001*
Al(T)	0.160	0.558	-0.861	0.766	0.814		0.732	0.596	0.849	0.536	0.643	0.401	0.610	0.509	0.203	-0.121	0.651	0.915	-0.484	-0.504	0.942	0.298	-0.633	0.829	0.901	-0.676	-0.862
	0.248	*<0.001*	*<0.001*	*<0.001*	*<0.001*		*<0.001*	*<0.001*	*<0.001*	*<0.001*	*<0.001*	*0.004*	*<0.001*	*<0.001*	*0.139*	*0.379*	*<0.001*	*<0.001*	*<0.001*	*<0.001*	*<0.001*	*0.030*	*<0.001*	*<0.001*	*<0.001*	*<0.001*	*<0.001*
Fe(T)	0.113	0.106	-0.616	0.476	0.799	0.734		0.699	0.637	0.172	0.174	0.160	0.226	0.162	0.160	-0.109	0.257	0.655	-0.052	-0.130	0.664	0.707	-0.342	0.818	0.761	-0.453	-0.611
	0.415	*0.446*	*<0.001*	*<0.001*	*<0.001*	*<0.001*		*<0.001*	*<0.001*	*0.210*	*0.205*	*0.244*	*0.100*	*0.238*	*0.244*	*0.427*	*0.061*	*<0.001*	*0.706*	*0.346*	*<0.001*	*<0.001*	*0.013*	*<0.001*	*<0.001*	*0.001*	*<0.001*
Mn(T)	0.339	0.075	-0.309	0.317	0.164	0.393	0.539		0.682	0.519	0.395	0.538	0.447	0.527	0.527	0.253	0.544	0.501	-0.185	-0.339	0.550	0.521	-0.191	0.410	0.457	-0.249	-0.408
	0.012	*0.592*	*0.023*	*0.020*	*0.235*	*0.003*	*<0.001*		*<0.001*	*<0.001*	*0.004*	*<0.001*	*0.001*	*<0.001*	*<0.001*	*0.066*	*<0.001*	*<0.001*	*0.179*	*0.014*	*<0.001*	*<0.001*	*0.165*	*0.003*	*0.001*	*0.070*	*0.003*
Cu(T)	0.361	0.638	-0.919	0.888	0.657	0.828	0.631	0.469		0.674	0.712	0.496	0.680	0.637	0.338	0.016	0.771	0.878	-0.463	-0.593	0.915	0.280	-0.544	0.651	0.816	-0.631	-0.691
	0.007	*<0.001*	*<0.001*	*<0.001*	*<0.001*	*<0.001*	*<0.001*	*<0.001*		*<0.001*	*<0.001*	*<0.001*	*<0.001*	*<0.001*	*0.014*	*0.906*	*<0.001*	*<0.001*	*0.001*	*<0.001*	*<0.001*	*0.042*	*<0.001*	*<0.001*	*<0.001*	*<0.001*	*<0.001*
OC	0.802	0.476	-0.502	0.607	-0.005	0.412	0.078	0.331	0.651		0.698	0.915	0.922	0.988	0.754	0.555	0.977	0.496	-0.277	-0.419	0.579	0.185	-0.016	0.059	0.391	-0.152	-0.351
	<0.001	*<0.001*	*<0.001*	*<0.001*	*0.973*	*0.002*	*0.575*	*0.015*	*<0.001*		*<0.001*	*<0.001*	*<0.001*	*<0.001*	*<0.001*	*<0.001*	*<0.001*	*<0.001*	*0.044*	*0.002*	*<0.001*	*0.179*	*0.910*	*0.669*	*0.004*	*0.269*	*0.011*
BC	0.346	0.704	-0.634	0.684	0.240	0.549	0.133	0.217	0.755	0.769		0.543	0.681	0.646	0.320	0.055	0.760	0.637	-0.582	-0.659	0.654	-0.149	-0.481	0.333	0.456	-0.497	-0.398
	0.010	*<0.001*	*<0.001*	*<0.001*	*0.080*	*<0.001*	*0.340*	*0.115*	*<0.001*	*<0.001*		*<0.001*	*<0.001*	*<0.001*	*0.020*	*0.691*	*<0.001*	*<0.001*	*<0.001*	*<0.001*	*<0.001*	*0.278*	*0.001*	*0.015*	*0.001*	*<0.001*	*0.004*
FA(SP)	0.899	0.172	-0.284	0.387	-0.134	0.287	0.122	0.466	0.422	0.861	0.443		0.854	0.940	0.851	0.722	0.882	0.302	-0.056	-0.229	0.383	0.362	0.234	-0.061	0.228	0.117	-0.172
	<0.001	*0.214*	*0.037*	*0.004*	*0.336*	*0.036*	*0.379*	*<0.001*	*0.002*	*<0.001*	*0.001*		*<0.001*	*<0.001*	*<0.001*	*<0.001*	*<0.001*	*0.028*	*0.683*	*0.096*	*0.005*	*0.008*	*0.088*	*0.658*	*0.097*	*0.396*	*0.212*

HA(SP)	0.492	0.579	-0.536	0.590	0.167	0.545	0.103	0.151	0.637	0.824	0.885	0.598		0.897	0.585	0.444	0.951	0.558	-0.279	-0.389	0.637	0.158	-0.102	0.183	0.490	-0.168	-0.470
	<0.001	<0.001	<0.001	<0.001	0.226	<0.001	0.460	0.274	<0.001	<0.001	<0.001	<0.001		<0.001	<0.001	0.001	<0.001	<0.001	0.042	0.005	<0.001	0.250	0.457	0.184	<0.001	0.221	0.001
HN(SP)	0.820	0.433	-0.469	0.582	-0.049	0.339	0.055	0.344	0.622	0.982	0.694	0.849	0.708		0.771	0.577	0.961	0.457	-0.221	-0.367	0.540	0.226	0.038	0.024	0.352	-0.092	-0.322
	<0.001	0.001	<0.001	<0.001	0.725	0.012	0.694	0.011	<0.001	<0.001	<0.001	<0.001	<0.001		<0.001	<0.001	<0.001	0.001	0.108	0.008	<0.001	0.100	0.781	0.863	0.010	0.504	0.019
FA(SH)	0.817	-0.200	0.044	0.049	-0.307	0.025	0.050	0.499	0.087	0.574	-0.006	0.865	0.241	0.596		0.849	0.678	0.084	0.118	-0.053	0.166	0.552	0.437	-0.195	0.073	0.168	0.025
	<0.001	0.147	0.752	0.723	0.024	0.857	0.719	<0.001	0.531	<0.001	0.964	<0.001	0.079	<0.001		<0.001	<0.001	0.543	0.389	0.700	0.226	<0.001	0.002	0.156	0.594	0.221	0.857
HA(SH)	0.754	-0.314	0.284	-0.201	-0.425	-0.161	-0.160	0.274	-0.173	0.429	-0.151	0.745	0.133	0.449	0.903		0.460	-0.284	0.241	0.154	-0.185	0.373	0.712	-0.498	-0.260	0.494	0.288
	<0.001	0.021	0.037	0.146	0.001	0.246	0.247	0.045	0.210	0.001	0.275	<0.001	0.339	0.001	<0.001		0.001	0.039	0.079	0.263	0.179	0.007	<0.001	<0.001	0.058	<0.001	0.036
HN(SH)	0.660	0.611	-0.614	0.704	0.107	0.483	0.107	0.256	0.744	0.967	0.884	0.720	0.866	0.941	0.355	0.191		0.612	-0.387	-0.524	0.686	0.185	-0.154	0.195	0.505	-0.253	-0.455
	<0.001	<0.001	<0.001	<0.001	0.442	<0.001	0.443	0.062	<0.001	<0.001	<0.001	<0.001	<0.001	<0.001	0.009	0.167		<0.001	0.005	<0.001	<0.001	0.179	0.263	0.156	<0.001	0.066	0.001
ECEC	0.072	0.688	-0.945	0.853	0.857	0.893	0.674	0.221	0.903	0.403	0.622	0.147	0.498	0.363	-0.197	-0.387	0.541		-0.523	-0.570	0.988	0.179	-0.767	0.858	0.953	-0.816	-0.888
	0.603	<0.001	<0.001	<0.001	<0.001	<0.001	<0.001	0.108	<0.001	0.003	<0.001	0.289	<0.001	0.007	0.154	0.004	<0.001		<0.001	<0.001	<0.001	0.194	<0.001	<0.001	<0.001	<0.001	<0.001
EA	0.138	-0.842	0.557	-0.608	-0.190	-0.351	-0.024	-0.202	-0.453	-0.345	-0.478	-0.051	-0.358	-0.346	0.158	0.201	-0.440	-0.482		0.935	-0.513	0.453	0.727	-0.320	-0.351	0.525	0.430
	0.319	<0.001	<0.001	<0.001	0.168	0.009	0.866	0.143	0.001	0.011	<0.001	0.716	0.008	0.010	0.253	0.146	0.001	<0.001		<0.001	<0.001	0.001	<0.001	0.020	0.011	<0.001	0.002
Al(EA)	-0.035	-0.748	0.570	-0.594	-0.279	-0.400	-0.174	-0.302	-0.506	-0.407	-0.440	-0.204	-0.382	-0.402	-0.019	0.035	-0.457	-0.510	0.928		-0.563	0.294	0.651	-0.305	-0.376	0.453	0.354
	0.801	<0.001	<0.001	<0.001	0.041	0.003	0.209	0.027	<0.001	0.002	0.001	0.139	0.004	0.003	0.889	0.799	0.001	<0.001	<0.001		<0.001	0.033	<0.001	0.027	0.006	0.001	0.010
PCEC	0.231	0.631	-0.940	0.855	0.831	0.938	0.729	0.332	0.930	0.503	0.639	0.307	0.554	0.460	-0.025	-0.231	0.603	0.979	-0.413	-0.478		0.214	-0.709	0.818	0.951	-0.763	-0.887
	0.093	<0.001	<0.001	<0.001	<0.001	<0.001	<0.001	0.014	<0.001	<0.001	<0.001	0.024	<0.001	0.001	0.855	0.094	<0.001	<0.001	0.002	<0.001		0.120	<0.001	<0.001	<0.001	<0.001	<0.001
PA	0.375	-0.559	0.020	-0.156	0.394	0.268	0.639	0.517	0.146	-0.053	-0.289	0.216	-0.200	-0.032	0.422	0.349	-0.175	0.076	0.471	0.249	0.199		0.309	0.307	0.352	0.075	-0.134
	0.005	<0.001	0.884	0.260	0.003	0.050	<0.001	<0.001	0.292	0.703	0.034	0.117	0.146	0.819	0.002	0.010	0.207	0.583	<0.001	0.070	0.149		0.025	0.026	0.010	0.583	0.328
Al(PA)	0.484	-0.739	0.702	-0.641	-0.614	-0.505	-0.316	0.022	-0.570	0.004	-0.453	0.368	-0.202	0.028	0.647	0.795	-0.215	-0.743	0.663	0.575	-0.613	0.366		-0.746	-0.674	0.847	0.721
	<0.001	<0.001	<0.001	<0.001	<0.001	<0.001	0.020	0.873	<0.001	0.976	0.001	0.006	0.142	0.842	<0.001	<0.001	0.118	<0.001	<0.001	<0.001	<0.001	0.007		<0.001	<0.001	<0.001	<0.001
SSA(aN_2)	-0.185	0.346	-0.698	0.514	0.979	0.811	0.761	0.141	0.637	-0.048	0.233	-0.194	0.112	-0.081	-0.379	-0.478	0.077	0.850	-0.216	-0.271	0.817	0.334	-0.645		0.907	-0.779	-0.828
	0.182	0.010	<0.001	<0.001	<0.001	<0.001	<0.001	0.310	<0.001	0.729	0.091	0.161	0.422	0.559	0.005	<0.001	0.581	<0.001	0.117	0.048	<0.001	0.014	<0.001		<0.001	<0.001	<0.001
SSA(aH_2O)	0.058	0.505	-0.843	0.700	0.946	0.898	0.747	0.214	0.838	0.281	0.509	0.070	0.407	0.237	-0.220	-0.373	0.406	0.964	-0.328	-0.394	0.953	0.265	-0.660	0.937		-0.767	-0.908
	0.679	<0.001	<0.001	<0.001	<0.001	<0.001	<0.001	0.121	<0.001	0.039	<0.001	0.616	0.002	0.085	0.110	0.006	0.002	<0.001	0.016	0.003	<0.001	0.053	<0.001	<0.001		<0.001	<0.001
r(dN_2)mean	0.520	-0.509	0.607	-0.531	-0.603	-0.481	-0.422	-0.049	-0.520	0.120	-0.343	0.422	-0.091	0.143	0.628	0.863	-0.094	-0.659	0.387	0.289	-0.554	0.191	0.894	-0.634	-0.616		0.797
	<0.001	<0.001	<0.001	<0.001	<0.001	<0.001	0.002	0.726	<0.001	0.386	0.011	0.002	0.512	0.303	<0.001	<0.001	0.499	<0.001	0.004	0.034	<0.001	0.166	<0.001	<0.001	<0.001		<0.001
r(dH_2O)mean	-0.121	-0.443	0.781	-0.681	-0.781	-0.849	-0.677	-0.085	-0.710	-0.298	-0.411	-0.126	-0.413	-0.249	0.135	0.235	-0.390	-0.835	0.215	0.198	-0.850	-0.200	0.460	-0.780	-0.844	0.464	
	0.383	0.001	<0.001	<0.001	<0.001	<0.001	<0.001	0.543	<0.001	0.029	0.002	0.364	0.002	0.069	0.330	0.088	0.004	<0.001	0.119	0.152	<0.001	0.148	0.001	<0.001	<0.001	<0.001	

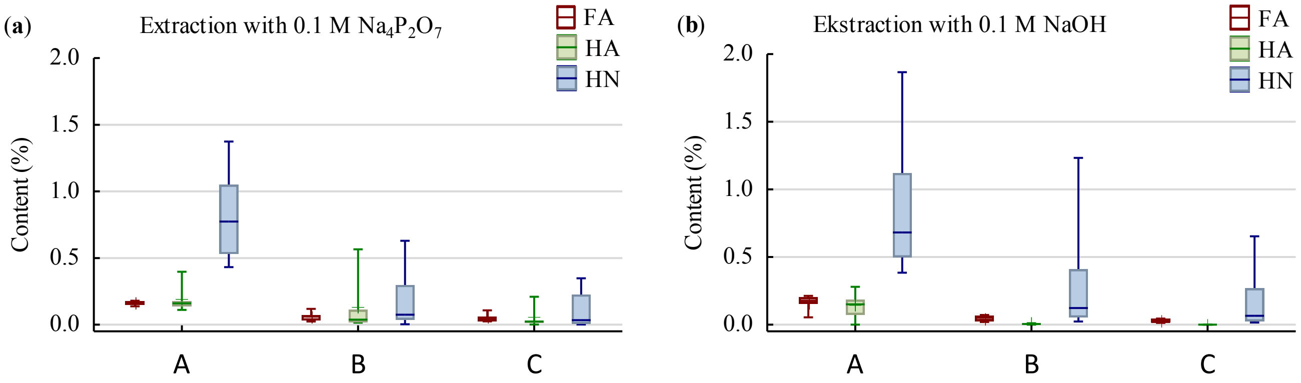

Figure S3. (**a**) Contents of *FA*, *HA*, and *HN* determined based on the extractions with 0.1 M $Na_4P_2O_7$ and (**b**) 0.1 M NaOH. The box plots show the minimum, first quartile, median, third quartile, and maximum. A – topsoils (10–15 cm), B – upper subsoils (45–50 cm), and C – lower subsoils (75–80 cm).

C. Elemental composition, FT-IR spectra, and adsorption in HF-altered soils.

The obtained absorbance spectra were analyzed using the OriginPro 2015 software. The relative intensities of the examined bands were estimated based on the baseline corrected peak areas. The baselines were created using the 1st and 2nd derivative, or the 2nd derivative methods.

The obtained in the above way "raw" intensities (I_r) of the individual bands are always burdened with errors arising during the sample preparation (differences in the particle sizes after grinding, at the actual ratio of the soil sample/KBr, or in density and homogeneity of pellets) and those related to the reproducibility of measurements. However, it was possible to correct these errors to a large extent. The PLSR indicated that *OC* in the altered soil samples (S4 Table) can be described using the following equation:

$$OC = 0.81 + 3.96 \cdot \text{V-2850}_{r\,(0.23,\,0.91)} + 2.89 \cdot \text{VI-1725}_{r\,(0.78,\,1.08)} \qquad R^2 = 0.919;\ LV = 2;\ Q^2 = 0.18 \qquad (S1)$$

where IV-2920$_r$ and VI-1725$_r$ denote the "raw" relative intensities of bands at 2920 and 1725 cm^{-1}, the values in brackets are their standardized regression coefficients and variable importance in projection (VIP), respectively. LV denotes the number of latent vectors, and Q^2 is the Wold's value for the second LV. Therefore, the corrected intensity of band X of soil *i* ($I(X)_i$) can be described as:

$$I(X)_i = \frac{OC_i}{0.81+3.96\cdot V-2850_{ri}+2.89\cdot VII-1725_{ri}} \cdot I_r(X)_i \qquad \text{(S2)}$$

where OC_i is the organic carbon content of soil i, and $IV\text{-}2850_{ri}$ and $VI\text{-}1725_{ri}$ are its "raw" relative intensities at 2850 and 1725 cm^{-1}, respectively. In such a way, the bands at 2850 and 1725 cm^{-1} were used as the internal standard of organic carbon content in each pellet.

Table S4. Elemental composition, corrected band intensities, $K_{d\,ac}$, $K_{d\,n}$, and pH values from the adsorption experiments in the HF-altered soils.

Property\Soil	A611Ap	L590Ap	L590Bt1	L564Ap	L564Bt1	L564Bt2	C587Ap	C587A2	C587AC	A805Ap	L824Ap	L824Bt1	L824Bt2
OC (%)[a]	16.15	11.77	1.45	7.43	4.78	2.07	10.58	9.65	5.00	11.78	7.83	2.18	1.67
N (%)	1.24	0.96	0.22	0.74	0.51	0.32	1.03	0.78	0.44	0.95	0.72	0.28	0.23
S (%)	0.13	0.12	0.04	0.10	0.07	0.02	0.12	0.06	0.05	0.11	0.10	0.10	0.07
H (%)	2.04	1.65	0.71	1.00	0.75	0.56	1.26	1.20	0.81	1.51	1.09	0.35	0.25
I-3700 (3687-3715 cm^{-1})[b]	0	0	1.31	0	0.03	0.22	0.03	0.08	0.08	0	0.05	0.03	0.05
II-3625 (3580-3682 cm^{-1})	0.09	2.33	9.45	1.28	1.90	3.58	1.31	1.88	1.08	0.50	1.73	4.31	1.44
III-3420 (3300-3583 cm^{-1})	27.22	12.22	11.57	18.45	22.92	9.29	12.98	11.14	30.28	22.18	25.65	9.96	5.16
IV-2920 (2879-3004 cm^{-1})	3.63	2.69	0.56	1.86	1.10	0.47	2.14	0.94	1.17	2.97	2.68	0.72	0.56
V-2850 (2828-2879 cm^{-1})	0.94	0.59	0.15	0.35	0.25	0.11	0.57	0.18	0.28	0.82	0.70	0.20	0.15
VI-1725 (1705-1790 cm^{-1})	4.02	3.02	0.04	1.75	1.07	0.36	2.49	2.83	1.04	2.62	1.51	0.28	0.15
VII-1640 (1571-1705 cm^{-1})	6.05	4.65	2.66	5.41	4.98	3.02	4.27	3.70	5.77	5.28	5.67	2.84	1.48
VIII-1535 (1503-1558 cm^{-1})	0.71	0.68	0.14	0.53	0.13	0.12	0.20	0.06	0.03	0.70	0.41	0.10	0.07
IX-1085 (1051-1449 cm^{-1})	60.48	125.93	210.37	133.75	174.09	165.85	129.68	156.81	165.83	107.25	230.53	131.52	83.13
X-1035 (843-1050 cm^{-1})	52.60	104.23	145.90	119.50	102.22	96.11	77.44	81.52	86.62	97.88	95.78	94.82	41.33
XI-795 (710-843 cm^{-1})	15.76	24.33	14.97	29.87	22.43	19.51	20.04	19.60	18.80	29.49	27.48	15.50	9.56
$K_{d\,ac}$ (mL/g)	437.4	231.5	16.9	139.1	90.4	26.3	194.5	261.5	67.3	245.6	159.2	25.0	18.8
(SD)	(28.7)	(5.7)	(0.6)	(9.8)	(2.1)	(5.5)	(11.3)	(18.8)	(3.1)	(11.3)	(5.9)	(0.7)	(1.6)
pH	2.8	2.9	3.6	2.9	2.9	3.3	2.7	2.5	3.1	2.9	2.9	3.6	3.7
$K_{d\,n}$ (mL/g)	135.5	97.6	5.0	51.9	25.8	3.4	66.8	54.0	25.5	78.3	82.3	17.1	8.9
(SD)	(13.5)	(5.2)	(0.4)	(4.3)	(0.4)	(1.3)	(10.2)	(12.6)	(2.5)	(0.9)	(5.2)	(0.9)	(0.4)
pH	7.0	6.8	7.3	7.2	6.6	7.4	6.4	6.1	6.8	7.1	7.0	6.7	6.8

[a] determined using a Vario El cube CHNS elemental analyzer, [b] mean integration ranges.

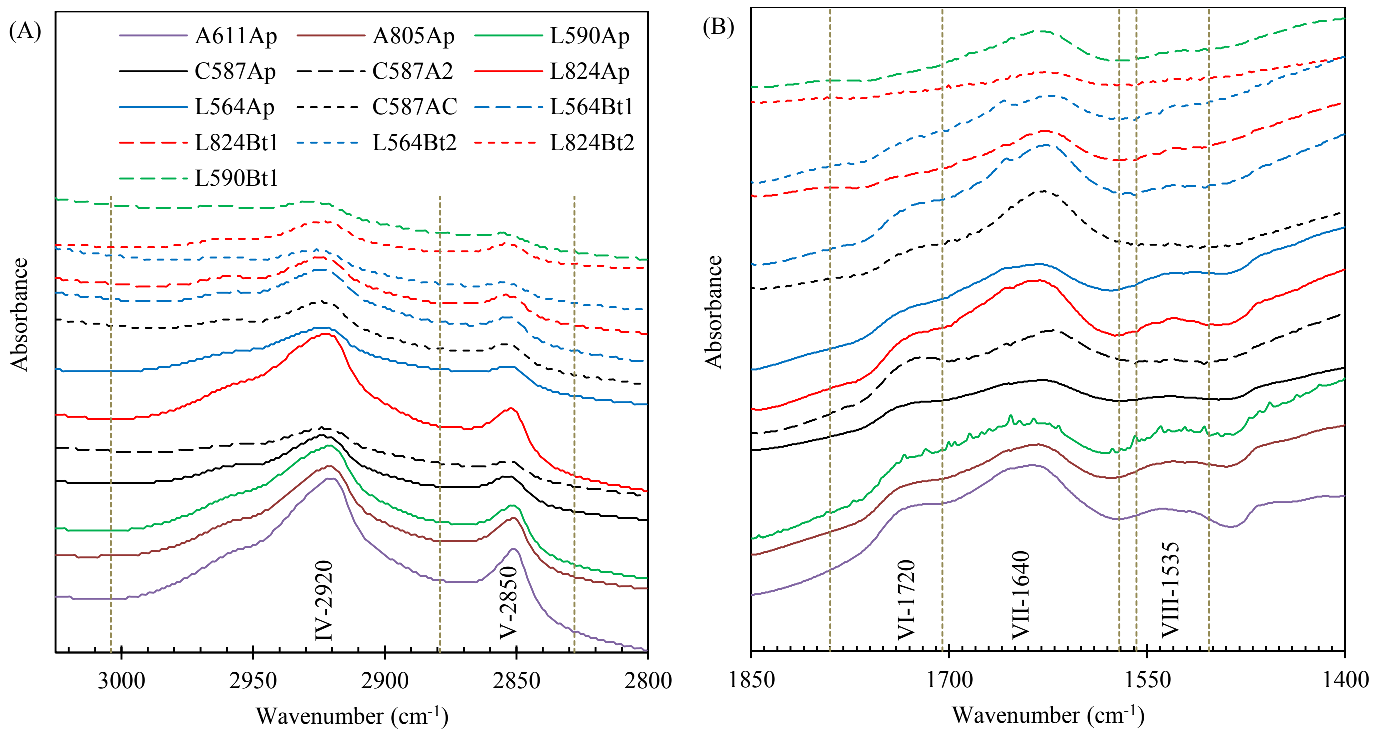

Figure S4. Selected ranges of FT-IR spectra of 13 HF-altered soils, shifted vertically to avoid overlapping and arranged in order from the largest (A611Ap) to the smallest (L590Bt1) *OC*. The vertical dashed lines indicate the mean integration ranges.

Table S5. Band assignments of the FT-IR spectra of HF altered soils. Notation of vibrational modes: ν-stretching, δ-bending, γ-out of plane bending, s-symmetric, a-asymetric.

No	Location (cm^{-1})	Main component	Other components	References
I	3700	kaolinite	n/a	[15-17]
II	3625	smectite; illite	n/a	[16, 17]
III	3420	$\nu(O–H)_{phenolic, alcohol}$	smectite; illite; $\nu(N–H)_{amine}$	[15, 16, 18]
IV	2920	$\nu_{as}(CH_2)_{aliphatic}$	n/a	[15, 18]
V	2850	$\nu_s(CH_2)_{aliphatic}$	n/a	[15, 18]
VI	1725	$\nu(C=O)_{carboxyl}$	$\nu(C=O)_{ester, carbonyl}$	[18-20]
VII	1640	$\nu(C=C)_{aromatic}$	smectite; illite; $\nu(C=O)_{amide}$; $\delta(N–H)_{amide, amine}$	[16-18]
VIII	1535	$\nu(C=C)_{aromatic}$	n/a	[18]

IX	1085	quartz	smectite; kaolinite; illite	[15-17]
X	1035	smectite; kaolinite; illite	δ(C–H)$_{aromatic}$	[15-17]
XI	795	γ(C–H)$_{heteroaromatic, polynyclear aromatic,}$	quartz	[18, 19]

D. Molecular structure of TB.

(a) TB

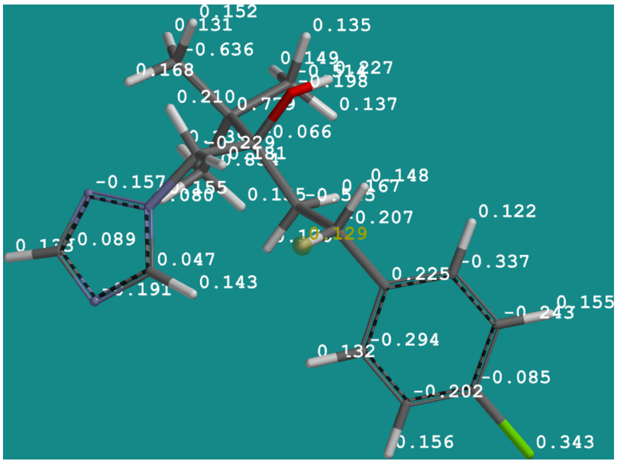

(b) TBH$^+$

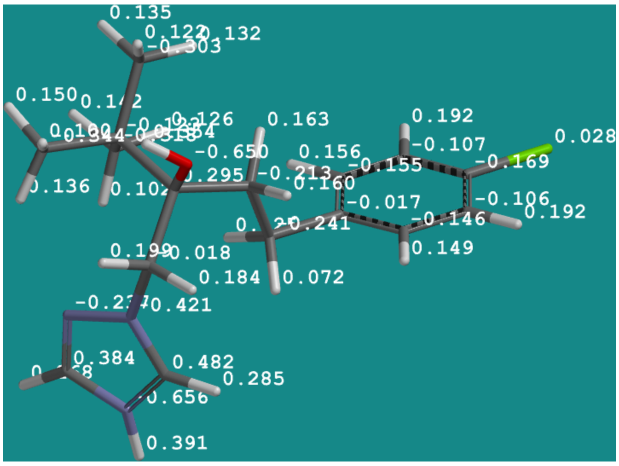

Figure S5. (**a**) The Mulliken charge distribution for molecular and (**b**) protonated at N(4) forms of TB. Calculations were made at the RHF 6-311+G** basis set.

References

1. ISO 11277, Soil quality – Determination of particle size distribution in mineral soil material – Method by sieving and sedimentation. 2020.
2. Cave, M. R.; Harmon, K., Determination of trace metal distributions in the iron oxide phases of red bed sandstones by chemometric analysis of whole rock and selective leachate data. *Analyst* **1997,** 122, 501-512. DOI: 10.1039/A607953I
3. Ramnarine, R.; Voroney, R. P.; Wagner-Riddle, C.; Dunfield, K. E., Carbonate removal by acid fumigation for measuring the δ ^{13}C of soil organic carbon. *Can. J. Soil Sci.* **2011,** 91, 247-250. DOI: 10.4141/CJSS10066
4. Schmidt, M. W. I.; Skjemstad, J. O.; Czimczik, C. I.; Glaser, B.; Prentice, K. M.; Gelinas, Y.; Kuhlbusch, A. J., Comparative analysis of black carbon in soils. *Global Biogeochem. Cy.* **2001,** 15, 163-167. DOI: 10.1029/2000GB001284
5. Fox, P. M.; Nico, P. S.; Tfaily, M. M.; Heckman, K.; Davis, J., Characterization of natural organic matter in low-carbon sediments: Extraction and analytical approaches. *Org. Geochem.* **2017,** 114, 12-22. DOI: 10.1016/j.orggeochem.2017.08.009
6. ISO 12782-4, Soil quality – Parameters for geochemical modelling of leaching and speciation of constituents in soils and materials – Part 4: Extraction of humic substances from solid samples. 2012.
7. ISO 11260, Soil quality – Determination of effective cation exchange capacity and base saturation level using barium chloride solution. 2018.
8. ISO 14254, Soil quality – Determination of exchangeable acidity using barium chloride solution as extractant. **2018**.

9. Shokrollahi, A.; Ghaedi, M.; Niband, M. S.; Rajabi, H. R., Selective and sensitive spectrophotometric method for determination of sub-micro-molar amounts of aluminium ion. *J. Hazard. Mater.* **2008,** 151, 642-648. DOI: 10.1016/j.jhazmat.2007.06.037
10. ISO 13536, Soil quality – Determination of the potential cation exchange capacity and exchangeable cations using barium chloride solution buffered at pH = 8.1. 1995.
11. Curtin, D.; Rostad, H. P. W., Cation exchange and buffer potential of Saskatchewan soils estimated from texture, organic matter and pH. *Can. J. Soil Sci.* **1997,** 77, 621-626. DOI: 10.4141/S97-015
12. Siek, M.; Paszko, T., Factors affecting coupled degradation and time-dependent sorption processes of tebuconazole in mineral soil profiles. *Sci. Total Environ.* **2019,** 690, 1035-1047. DOI: 10.1016/j.scitotenv.2019.06.409
13. Ukalska-Jaruga, A.; Smreczak, B.; Klimkowicz-Pawlas, A., Soil organic matter composition as a factor affecting the accumulation of polycyclic aromatic hydrocarbon. *J. Soils Sediments* **2019,** 19, 1890-1900. DOI: 10.1007/s11368-018-2214-x
14. Giles, C. H.; MacEwan, T. H.; Nakhwa, S. N.; Smith, D., 786. Studies in adsorption. Part XI. A system of classification of solution adsorption isotherms, and its use in diagnosis of adsorption mechanisms and in measurement of specific surface areas of solids. *J. Chem. Soc.* **1960**, 3973-3993. DOI: 10.1039/JR9600003973
15. Krivoshein, P. K.; Volkov, D. S.; Rogova, O. B.; Proskurnin, M. A., FTIR photoacoustic spectroscopy for identification and assessment of soil components: Chernozems and their size fractions. *Photoacoustics* **2020,** 18, 100162. DOI: 10.1016/j.pacs.2020.100162
16. Chukanov, N. V., *Infrared spectra of mineral species. Extended library. Vol. 1*. Springer: Dordrecht, 2014; p 1726.
17. Müller, C. M.; Pejcic, B.; Esteban, L.; Delle Piane, C.; Raven, M.; Mizaikoff, B., Infrared attenuated total reflectance spectroscopy: an innovative strategy for analyzing mineral components in energy relevant systems. *Sci. Rep.* **2014,** 4, 6764. DOI: 10.1038/srep06764
18. Silverstein, R. M.; Webster, F. X.; Kiemle, D. J., *Spectrometric identification of organic compounds. Seventh edition.* John Wiley & Sons, INC.: United States, 2005; p 502.
19. Tatzber, M.; Stemmer, M.; Spiegel, H.; Katzlberger, C.; Haberhauer, G.; Mentler, A.; Gerzabek, M. H., FTIR-spectroscopic characterization of humic acids and humin fractions obtained by advanced NaOH, $Na_4P_2O_7$, and Na_2CO_3 extraction procedures. *J. Plant Nutr. Soil Sc.* **2007,** 170, 522-529. DOI: 10.1002/jpln.200622082
20. Plaza, C.; Senesi, N.; Polo, A.; Brunetti, G., Acid-base properties of humic and fulvic acids formed during composting. *Environ. Sci. Technol.* **2005,** 39, 7141-7146. DOI: 10.1021/*es050613h*
